# Industrial Development of Standardized Fetal Progenitor Cell Therapy for Tendon Regenerative Medicine: Preliminary Safety in Xenogeneic Transplantation

**DOI:** 10.3390/biomedicines9040380

**Published:** 2021-04-03

**Authors:** Alexis Laurent, Philippe Abdel-Sayed, Anthony Grognuz, Corinne Scaletta, Nathalie Hirt-Burri, Murielle Michetti, Anthony S. de Buys Roessingh, Wassim Raffoul, Peter Kronen, Katja Nuss, Brigitte von Rechenberg, Lee Ann Applegate, Salim E. Darwiche

**Affiliations:** 1 Regenerative Therapy Unit, Lausanne University Hospital, University of Lausanne, CH-1066 Épalinges, Switzerland; alexis.laurent@unil.ch (A.L.); philippe.abdel-sayed@chuv.ch (P.A.-S.); anthony.grognuz@bluewin.ch (A.G.); corinne.scaletta@chuv.ch (C.S.); nathalie.burri@chuv.ch (N.H.-B.); murielle.michetti@chuv.ch (M.M.); lee.laurent-applegate@chuv.ch (L.A.A.); 2 Preclinical Research Department, LAM Biotechnologies SA, CH-1066 Épalinges, Switzerland; 3 Manufacturing Department, TEC-PHARMA SA, CH-1038 Bercher, Switzerland; 4 Children and Adolescent Surgery Service, Lausanne University Hospital, University of Lausanne, CH-1011 Lausanne, Switzerland; anthony.debuys-roessingh@chuv.ch; 5 Plastic, Reconstructive, and Hand Surgery Service, Lausanne University Hospital, University of Lausanne, CH-1011 Lausanne, Switzerland; wassim.raffoul@chuv.ch; 6 Musculoskeletal Research Unit, Vetsuisse Faculty, University of Zurich, CH-8057 Zurich, Switzerland; peter.kronen@vas-int.com (P.K.); katja.nuss@vetclinics.uzh.ch (K.N.); brigitte.vonrechenberg@uzh.ch (B.v.R.); 7 Center for Applied Biotechnology and Molecular Medicine, University of Zurich, CH-8057 Zurich, Switzerland; 8 Oxford OSCAR Suzhou Center, Oxford University, Suzhou 215123, China

**Keywords:** cell banking, chorioallantoic membrane model, fetal progenitor cell therapy, pilot safety study, preclinical animal model, regenerative medicine, tendinopathies, tendon cell therapy, toxicity evaluation, transplantation program

## Abstract

Tendon defects require multimodal therapeutic management over extensive periods and incur high collateral burden with frequent functional losses. Specific cell therapies have recently been developed in parallel to surgical techniques for managing acute and degenerative tendon tissue affections, to optimally stimulate resurgence of structure and function. Cultured primary human fetal progenitor tenocytes (hFPT) have been preliminarily considered for allogeneic homologous cell therapies, and have been characterized as stable, consistent, and sustainable cell sources in vitro. Herein, optimized therapeutic cell sourcing from a single organ donation, industrial transposition of multi-tiered progenitor cell banking, and preliminary preclinical safety of an established hFPT cell source (i.e., FE002-Ten cell type) were investigated. Results underlined high robustness of FE002-Ten hFPTs and suitability for sustainable manufacturing upscaling within optimized biobanking workflows. Absence of toxicity or tumorigenicity of hFPTs was demonstrated in ovo and in vitro, respectively. Furthermore, a 6-week pilot good laboratory practice (GLP) safety study using a rabbit patellar tendon partial-thickness defect model preliminarily confirmed preclinical safety of hFPT-based standardized transplants, wherein no immune reactions, product rejection, or tumour formation were observed. Such results strengthen the rationale of the multimodal Swiss fetal progenitor cell transplantation program and prompt further investigation around such cell sources in preclinical and clinical settings for musculoskeletal regenerative medicine.

## 1. Introduction

Drastic modifications within rapidly evolving lifestyles (e.g., sedentary occupation, high-risk sports, long-term pharmacologic drug use) have continuously been driving the need for innovative therapeutic solutions in musculoskeletal medicine in recent years. This trend has been particularly important for structural repair and functional restoration of tendons [1,2,3,4]. Increasing interest around regenerative medicine has prompted widespread efforts toward preclinical and clinical research to eventually provide physicians and patients with effective management strategies for debilitating wounds or tissue defects [5,6,7]. Therein, additive or synergistic combinations of surgical intervention and cell-based therapies may prove to be essential for optimization of clinical outcomes. Due to specificities of tendon tissues (i.e., vascularization, dynamic force transmission, collagen fiber micro- and macro-structures), many considered therapeutic approaches to date have fallen short of clinician and patient expectations with regard to healing outcomes [8,9,10,11].

Indeed, imperfect inherent healing capacities, iatrogenesis, and diverse tissue disorders (e.g., calcification, lipoid degeneration, tendinosis) are common and characteristic burdens which have made therapeutic management of defective tendon tissues complex [12,13,14]. Relatively highly prevalent afflictions (i.e., acute or degenerative) often result in functional losses, disability, chronic pain, and productivity deficits in athletes and manual workers. Thereby, these afflictions necessitate highly specialized professional care and incur high burdens for public healthcare systems [15,16,17,18,19,20,21,22]. Elevated rates of secondary tendon ruptures and adhesions remain as major clinical concerns, resulting in formation of non-functional scar tissue [23]. Furthermore, delayed inflammatory reactions, low baseline metabolism, limited extracellular matrix (ECM) deposition, and complex structural reorganization or alignment of tendons limit the scope and efficacy of specific therapeutic interventions [20,24]. Transplantation techniques such as tendon transfers (e.g., surgical harvest and grafting of autologous vestigial tendons) have been effectively limited by rapid graft degeneration. Therefore, surgical approaches would most probably benefit from additional stimulation by appropriate therapeutic cells, for optimal elasticity, mobility, and tensile strength restoration [25,26,27,28].

Considering bioengineering approaches for tendon repair or regeneration promotion, decellularized cadaveric scaffold tissues (i.e., human, equine origins) have been shown to optimally conjugate biocompatibility, appropriate mechanical properties, and propensity toward therapeutic cell loading [29,30,31,32,33,34,35,36]. Arrays of potential cellular sources have therefore been proposed in tendon bioengineering workflows, comprising various stem cells, placenta cells, amniotic cells, platelet-derivatives, tendon sheath fibroblasts, and adult tenocytes. However, results have been variable and sometimes contradictory [37,38,39,40,41,42,43,44,45,46,47]. Recently, cultured primary human fetal progenitor tenocytes (hFPT) have been preliminarily investigated within allogeneic homologous regenerative medicine approaches of tendon ruptures (e.g., hand, Achilles, or rotator cuff tendons) or specific inflammatory diseases. Such novel therapeutic cell candidates were proposed with the goal of potentially mediating scarless tissue repair or regeneration [36,48,49]. These cell sources have been shown to present high technical and therapeutic potential in preclinical research. Indeed, advantages of hFPTs comprise the limited need for a single organ donation, high robustness and sustainability of multi-tiered banking workflows, stable karyotypic and tenogenic properties (i.e., type I collagen, scleraxis, and tenomodulin production) in vitro, and maintenance of a tissue-specific phenotype [49,50,51,52,53,54,55]. Additionally, stimulatory potential of in vitro adult tenocyte proliferation and maintenance of hFPT viability in appropriate hyaluronic acid (HA) hydrogel scaffolds have been demonstrated [48,49]. Importantly, it was projected that extensive and consistent hFPT cell banks could potentially be established and serve for the manufacture of over 10^8^ cell therapy product (i.e., standardized transplant or combined advanced therapy medicinal product) units. Following industry best practices, this objective is tangibly achievable after one single organ donation under the federally registered Swiss fetal progenitor cell (FPC) transplantation program [49]. Therein, development of off-the-freezer standardized transplant preparations (e.g., hFPTs in acellularized tissue scaffolds or in versatile hydrogels) for localized tissue replacement or tendon regeneration stimulation (e.g., in degenerative diseases, small hand injuries, fissures, or partial ruptures) have been the objective of recent translational research [37]. Injectable standardized transplants (i.e., viable hFPTs in HA-based hydrogels) have previously been developed and characterized, yielding adequate formulation properties for tendon repair applications (i.e., injectability, cell viability maintenance, rheological behaviour) [48]. Such approaches represent novel management options and aim to complement or replace alternatively proposed therapies or products available for tendon repair or regeneration [56,57,58,59].

Therefore, as we had previously shown, properly sourced, isolated, and cultured hFPTs uphold the quality standards required from active pharmaceutical ingredients (API) for regenerative medicine product development and implementation [49]. Furthermore, integrative frameworks such as transplantation programs for FPC type establishment guarantee the highest level of quality and safety for subsequent clinical applications [60,61,62,63,64,65]. Indeed, thorough validation of identity, purity, sterility, stability, safety, and efficacy of biological raw and starting materials may be robustly performed within industrial multi-tiered cell banking of hFPTs following good manufacturing practices (GMP) [64,65,66]. Such approaches have been historically validated notably in the field of vaccine substrate development and use, wherein biotechnological process optimization has been a cornerstone in the industrial manufacture of many currently marketed biologicals and vaccines. Adaptation of starting material sourcing and banking of isolated primary cell types therefore serve as thoroughly validated technical bases for exploitation of tissue-specific cell types within homologous therapeutic product development approaches [65].

Herein, optimized primary therapeutic FPC type establishment from a single organ donation, industrial transposition of multi-tiered cell banking, and preliminary safety of a clinical-grade enzymatically isolated hFPT cell source (i.e., FE002-Ten cell type) were extensively investigated and further documented, building on our previous reports [48,49]. The overall objectives comprised experimental confirmation of the validity of the devised and stringently optimized multi-tiered FPC biobanking workflows (i.e., robustness of hFPT manufacture upscaling). Objectives further comprised preliminary demonstration of the absence of toxicity and safety of the considered cell source in a standardized chorioallantoic membrane (CAM) model, in soft agar transformation assays, and in a lagomorph (i.e., white rabbits) model of patellar tendon partial-thickness defect, respectively. Specifically, in vitro hFPT lifespans and behaviour in large-scale culture expansions were assessed, in view of maximizing manufacturing yields within acceptable technical specifications. Original industrial transposition and upscaling workflows in view of therapeutic products or processes development and optimization following a single fetal organ donation (e.g., FE002, 2009) have been modelled and developed. Moreover, analysis and scoring of in vivo effects of clinically relevant hFPT doses formulated in HA-based hydrogels (i.e., injectable standardized transplant prototypes) enabled preliminary preclinical evaluation of treatment tolerance and potential early adverse effect occurrence in a functional wound healing environment. Studies such as those described herein strengthen the rationale set forth under the multimodal Swiss FPC transplantation program. Specifically, such research prompts continued investigation around the therapeutic potential of standardized cell sources in preclinical and clinical settings for musculoskeletal regenerative medicine.

## 2. Materials and Methods

### 2.1. Primary hFPT Isolation, Cell Banking, and Characterization

#### 2.1.1. Fetal Tendon Tissue Donation and Primary hFPT Enzymatic Isolation

The primary FPC source considered in the present research and used for the in vitro, in ovo, and in vivo preclinical toxicity and safety studies was the enzymatically isolated FE002-Ten cell type, one of the hFPT sources established after optimized multimodal processing of a fetal Achilles tendon sample (i.e., controlled pregnancy termination at 14 weeks of gestation) in 2009. Therein, FE002-Ten primary hFPTs were isolated using two tissue processing methodologies (i.e., enzymatic and non-enzymatic FPC isolation) from a voluntary organ donation (i.e., under Swiss law) and were subsequently mitotically propagated under appropriate in vitro inducive conditions (Supplementary Materials, Figures S1–S4). Briefly, for enzymatic FPC isolation, part of the biopsied tendon tissue was isolated and rinsed in conserved phosphate-buffered saline (PBS). The available tissue sample was then dissected into <0.03 mm^3^ fragments and placed in sterile centrifuge tubes (50 mL, Falcon^®^, Corning, Glendale, Arizona, USA) before being covered with 5 mL of warmed (i.e., 37 °C) trypsin-EDTA (i.e., 0.25% trypsin and 0.1% ethylenediaminetetraacetic acid, Gibco^™^, Thermo Fisher Scientific, Waltham, MA, USA) (Figure S3). After 15 min of incubation at 37 °C, enzymatic cell dissociation was ended by addition of initial growth medium containing fetal bovine serum (FBS). Dissociation tubes were then centrifuged at (230 ± 10) × *g* at ambient temperature for 15 min, before resulting cellular materials were resuspended in warmed initial growth medium (Figure S3). After cell enumeration and viability assessment, seeding of cell suspensions in culture Petri dishes, primary expansion, passage, secondary expansion of Passage 1 cells in culture flasks, cell harvest, and freezing were performed (Supplementary Materials, Figure S4). The considered cryopreserved material was defined as the enzymatically isolated FE002-Ten parental cell bank (PCB, Passage 1). Detailed methods and cell isolation workflows are presented in Supplementary Materials (Figures S1–S4).

#### 2.1.2. Pilot hFPT Expansion for Technical Specification Optimization

For preliminary assessment of overall quality (i.e., validation of PCB materials, culture condition optimization, consistency evaluation) of the enzymatically isolated FE002-Ten cell type, a pilot serial in vitro expansion campaign was devised. Therein, PCB materials were used in a recovery procedure. Frozen vials were transported to the production suite and rapidly thawed in a water bath set at 37 °C ± 2 °C until disappearance of all ice crystals. Cell suspensions were then transferred to centrifuge tubes and slowly diluted (i.e., dropwise medium addition) to reach a final concentration of 2.5 × 10^5^ cells/mL. Resulting cell suspensions were centrifuged at (230 ± 10) × *g* for 10 min at ambient temperature. Viable cell counts were manually determined using Trypan blue exclusion dye and the cutoff value for inclusion of cellular materials in the subsequent experiments was set at 85% relative viability upon initiation. The stock cell suspension then served for serial expansions performed in a variety of culture vessel types (i.e., manufacturers, vessel surfaces) using various sources of fetal bovine serum (i.e., manufacturers, lot numbers), in view of optimizing manufacturing yields and in vitro cell type lifespan, while preserving adequate cellular morphology and behavior. Once the optimal manufacturing parameters were determined, FE002-Ten cells were serially expanded up to Passage 12. At each passage procedure, part of the cell suspension was used to seed new culture vessels, while the remaining cells were cryopreserved and stored.

#### 2.1.3. Optimized Multi-Tiered hFPT Biobanking Workflow Elaboration

Specific characteristics of the enzymatically isolated FE002-Ten cell type yielding impact on manufacturing quality and efficiency were studied, such as evolutive cellular morphology, expansion kinetics, and total in vitro lifespan (i.e., between Passages 1 and 12). Characterization experiments were performed in triplicate, with three experimental repetitions. At all considered passages for characterization purposes, cell cultures were harvested for enumeration at approximately 80% confluency. Evolutive population doubling times (PDTs) were calculated. Following optimized and validated technical specifications, progeny tiered cell banks were thereafter derived from the original FE002-Ten PCB materials as described hereabove and established after appropriate serial culture expansions. Therein, hFPT master cell bank (MCB) lots were manufactured at Passage 2 following expansion in T75 flasks (Nunc^™^, Thermo Fisher Scientific, Waltham, MA, USA), using relative viable seeding densities of 1.5 × 10^3^ ± 100 cells/cm^2^. Subsequently and by analogy, working cell bank (WCB) lots at Passages 3–7 and end of production cell bank (EOPCB) lots at Passage 12 were manufactured. For quality assurance, each WCB lot was sampled (i.e., start, middle, and end of batches) and tested for sterility, mycoplasma absence, cell recovery, growth characteristics, and cell morphology. Therefore, corresponding batch records and certificates of analysis were established, summarizing lot composition, vial identifiers, manufacturing date, testing, specifications (i.e., sterility, cellular behavior, quantitative parameters), results, and release. Out-of-specification lots were discarded. All vials were referenced in a centralized master-log for comprehensive traceability.

### 2.2. In Vitro Tumorigenicity Study of hFPTs in Soft Agar Colony Formation Assays

A standard soft agar colony formation assay (i.e., transformation assay) was used to assess the potential of FE002-Ten hFPTs to grow in non-adherent settings and to form colonies in suspension. Microplates (i.e., 12-well and 24-well) served as experimental scaffolds. The solid layer of the assay was composed of 0.6% agarose (i.e., low-melting point agarose, Sigma-Aldrich^®^, St. Louis, MI, USA) in PBS and complete growth medium supplemented with 1% penicillin-streptomycin (Gibco™, Thermo Fisher Scientific, Waltham, MA, USA). The soft layer of the assay was composed of 0.4% agarose obtained as described hereabove, with the investigated cellular materials (i.e., in various concentrations of hFPTs ranging from 125 to 10^3^ cells/well) being suspended therein. Both layers were sequentially prepared and were of equal volumes. Tested items were FE002-Ten cells freshly harvested from confluent cultures. Positive control cells were HeLa and HCT 116 cancerous cell lines, freshly harvested from confluent cultures. Complete growth medium with penicillin-streptomycin was added on top of the agarose layers, and plates were incubated at 37 °C under 5% CO_2_ in a humidified incubator for 12 days. Cultures were regularly microscopically assessed. At the end of the assay, representative imaging was performed to assess the formation of non-adherent colonies. Colony size was visually assessed and recorded based on standard hemocytometer grids for positive control cell lines.

### 2.3. Preclinical Toxicity Study of hFPTs in a Standardized CAM Model

A standardized CAM model was devised to assess potential toxicity of FE002-Ten hFPTs and to study the in ovo effects thereof. In total, 60 fertilized chicken eggs (Animalco AG, Staufen, Switzerland) were incubated at 37 °C in a humidified hatching incubator (FIEM srl, Guanzate, Italy). The eggs were sequentially opened for exposition of the CAM (Figure S5). The reason for the delay between small and large opening creation was to allow optimal membrane detachment from the outer eggshell (i.e., possible with the small hole) and subsequently to obtain a homogenous and large exposed membrane surface for the assay. The two-step treatment of the eggshell is of prime importance to not disturb and destroy membrane (i.e., tissue and vasculature) integrity at the time of eggshell opening. After 11 days of initial incubation, 12 mm diameter O-rings (i.e., circular and full cross-section, white PTFE, Trelleborg Sealing Solutions Switzerland SA, Crissier, Switzerland) were placed on each CAM surface. On the 12th day, CAMs were photographically recorded, and test-items (i.e., 2.4 × 10^6^ FE002-Ten hFPTs harvested at Passages 6 and 12, reconstituted in 250 µL normal saline) or reference items (i.e., 250 µL normal saline) were dispensed in each O-ring. After inoculation with test or reference items, the eggs were parafilm-sealed and incubated as described hereabove. Then, 2 days later, viability assessments were performed, before photographic imaging on a fluorescence stereomicroscope (Leica M205 FA, Leica Camera AG, Wetzlar, Germany) was performed for each egg. Quality of angiogenesis was also comparatively assessed, along with observations on general egg appearance. Detailed assay methodology is presented in Supplementary Materials (Figure S5).

### 2.4. Preclinical Safety Study of hFPTs in a Rabbit Model of Patellar Tendon Defect

#### 2.4.1. GLP Pilot Safety Study Design

**Figure 1:**
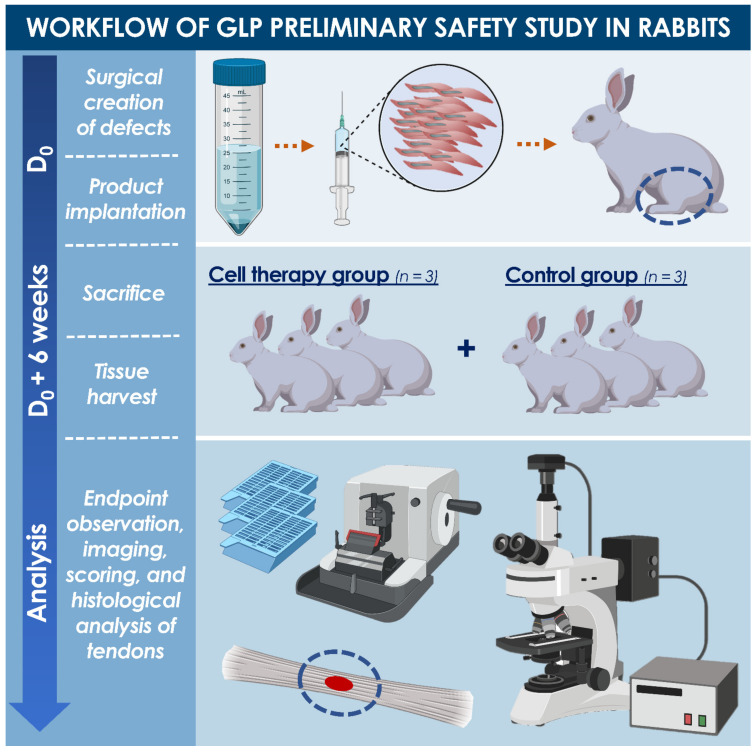
Workflow of the GLP preliminary safety study of hFPTs performed on a rabbit model of partial-thickness patellar tendon defect. On the day of defect creation, viable hFPTs were suspended in hyaluronic acid gels and delivered into the tendon defect, before suturing it closed, as well as the skin. A total of 6 weeks later, all rabbits were sacrificed, and tendons of interest were macroscopically assessed and harvested for histological processing and macroscopic evaluation, looking at potential early local adverse effects. GLP, good laboratory practice; hFPT, human fetal progenitor tenocytes.

A rabbit model was selected and adapted for the present study based on strong similarities between rabbit and human tendon tissues. All experiments were conducted in compliance with Swiss federal laws on animal protection and welfare and had been duly authorized by the Zurich cantonal Ethics Committee (License #ZH034/15). A total of six New Zealand white rabbits were included in the pilot safety study, performed in compliance with good laboratory practice (GLP), and were randomized to form two treatment groups (Figure 1). One group was operated and treated with the test-item (i.e., approximately 2.4 × 10^6^ hFPTs suspended in hyaluronic acid, Ostenil^®^ Tendon, TRB Chemedica AG, Feldkirchen, Germany) and the other group received the reference item (i.e., PBS diluted in hyaluronic acid). The chosen delivery scaffold (i.e., commercially available medical device for managing tendon-related pain) was composed of high molecular weight sodium hyaluronate (i.e., 2% hydrogel in isotonic buffer, with added mannitol, delivered prefilled in a sterile syringe). Appropriate quality controls were performed on manufactured products to verify cellular viability after extrusion (Supplementary Materials, Figure S9). All rabbits were operated on one hindlimb (i.e., according to randomization, Table A1), the other hindlimb serving as an unoperated and untreated internal control. Rabbits were sacrificed 6 weeks after surgery and the tendons of interest were harvested for evaluation and scoring, along with blood samples. Detailed GLP study methodology is presented in Supplementary Materials. Rabbit cadaver studies for model design optimization had been performed prior to initiation of the GLP pilot study (Figures S7 and S8).

#### 2.4.2. GLP Pilot Safety Study Workflow

Animals were acclimatized at least 14 days prior to surgery. Animals were then sedated, an intravenous catheter was placed, and anesthesia was induced. A blood sample was collected at that moment. Then, 3 cm-long incisions were created on the lateral face of the knee, extending to the medial face from 1 cm above to 2 cm below the knee joint space (Figure S6).

A mid-tendon scalpel incision was made in the uppermost layer of the patellar tendon, creating a flap (i.e., approximately 6 mm long and 2–2.5 mm wide). The flap was carefully lifted, and a hollow space was created with a microscalpel blade within the underlying tendon tissue, preserving the outer wall of the tendon. The flap was then partially suture-closed with a resorbable suture material and the test or reference products were delivered into the defect cavity, filling it completely. The tendon flap was then completely sutured closed. The fascia was closed in layers in a simple continuous fashion, the skin was closed, and wounds were covered with bulky dressing without additional immobilization. Post-operative analgesia was administered, along with prophylactic post-operational antibiotic therapy. Animals were housed in individual cages until fully recovered from the intervention (i.e., 24 to 72 h), then reintroduced into group housing. The animals were monitored twice daily, assessing alertness, appetite, posture, pain, and lameness. Then, 6 weeks post-operatively, animals were sedated, an intravenous catheter was placed, the final blood sampling was performed, then the animals were euthanized with pentobarbital (i.e., 0.5 mL/kg_BW_ i.v.).

#### 2.4.3. Processing of Harvested Tendons

The treated patellar tendons were cut at the insertion of the tuberositas tibiae and patella, inspected macroscopically, and documented using digital photography. Special focus was placed on key indicators such as local inflammation, tissue adhesion, fibrosis, and global tissue quality. A specific scoring method was devised, and scoring was performed to semi-quantitatively evaluate the tissues (Table A2). Harvested tendons were then transferred to 4% formalin, before being processed for histological analysis. The contralateral untreated patellar tendons were also harvested and placed in 4% formalin, to serve as untreated controls. Tissues were subsequently placed in 75% ethanol for up to three days before embedding in paraffin. Sections of 5 µm were cut in the sagittal plane following the direction of tendon fibers and were stained with hematoxylin-eosin, Alcian Blue, Picrosirius Red, and von Kossa stains. Preliminary and early tendon healing was assessed by studying tendon morphology at the original defect site. The defect location, size, and edges were observed and graded, along with the reactivity of the tissue surface (Table A2). Specific zones were then selected within the defect areas for microscopic evaluation under a 10× objective and scoring was performed again (Table A3). In situ hybridization was performed on tissue sections for the detection human DNA ALU repeats. Detailed methodology is presented in Supplementary Materials (Figures S10–S12).

#### 2.4.4. Statistical Analysis

Fisher’s Test for exact count data (i.e., two-sided, astatsa.com/FisherTest/, by N. Vasavada, 2016) was performed for each parameter of the microscopic evaluation of harvested and processed tendon tissues and results were summarized in a condensed contingency table. Based on obtained results, independency between investigated parameters and study groups was confirmed or excluded. The significance cut-off was set at *p* < 0.05.

## 3. Results

### 3.1. Optimized Technical Specifications for Multi-Tiered hFPT Cell Banking

**Figure 2:**
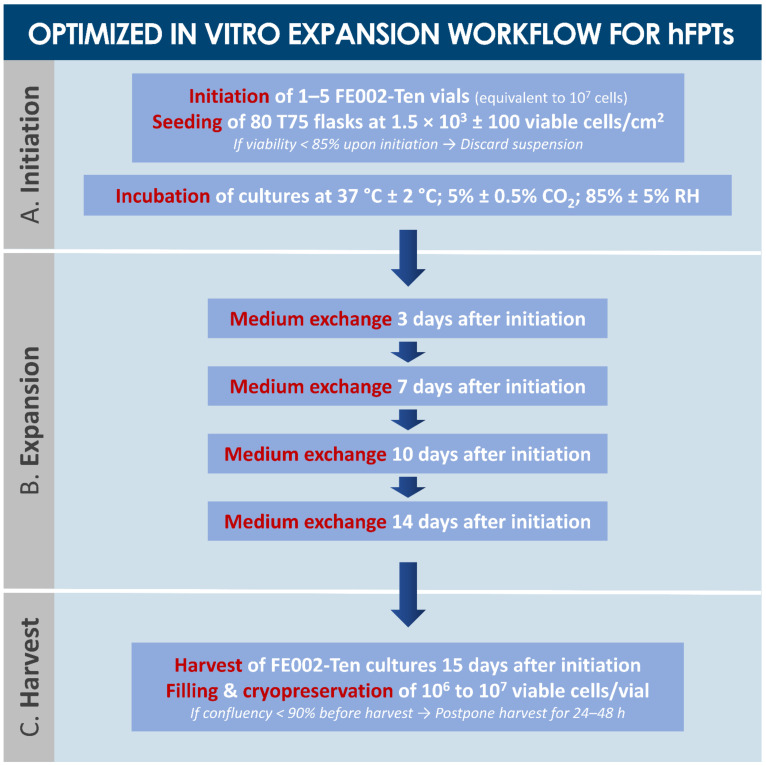
Optimized in vitro expansion workflow for large-scale banking of FE002-Ten hFPTs. Multiple optimization steps and parameter validation (i.e., cell seeding densities, culture periods, choice of serum source and culture surface type) enabled the selection of adapted culture conditions and related technical specifications for maximization of manufacturing efficiency of homogenous materials. The presented workflow was applicable for Passages 1 to 8, which are of interest for clinical applications (i.e., higher passages were characterized by longer PDTs and required an additional medium exchange step). Complete culture medium was composed of Dulbecco’s Modified Eagle Medium, supplemented with 10% *v/v* FBS and L-glutamine. Cryopreservation medium was composed of 50% *v/v* complete medium, 40% *v/v* FBS, and 10% *v/v* DMSO. Optimal proliferation conditions were obtained with a clinical-grade Sigma^®^ FBS lot, using 75 cm^2^ culture flasks, cell seeding at 1.5 × 10^3^ ± 100 viable cells/cm^2^ (**A**), culture medium volumes of 10 mL/flask, culture medium exchanges twice/week (**B**), and 14–15 days of culture before harvest (**C**). DMSO, dimethyl sulfoxide; FBS, fetal bovine serum; hFPT, human fetal progenitor tenocytes; PDT, population doubling time; RH, relative humidity.

**Figure 3:**
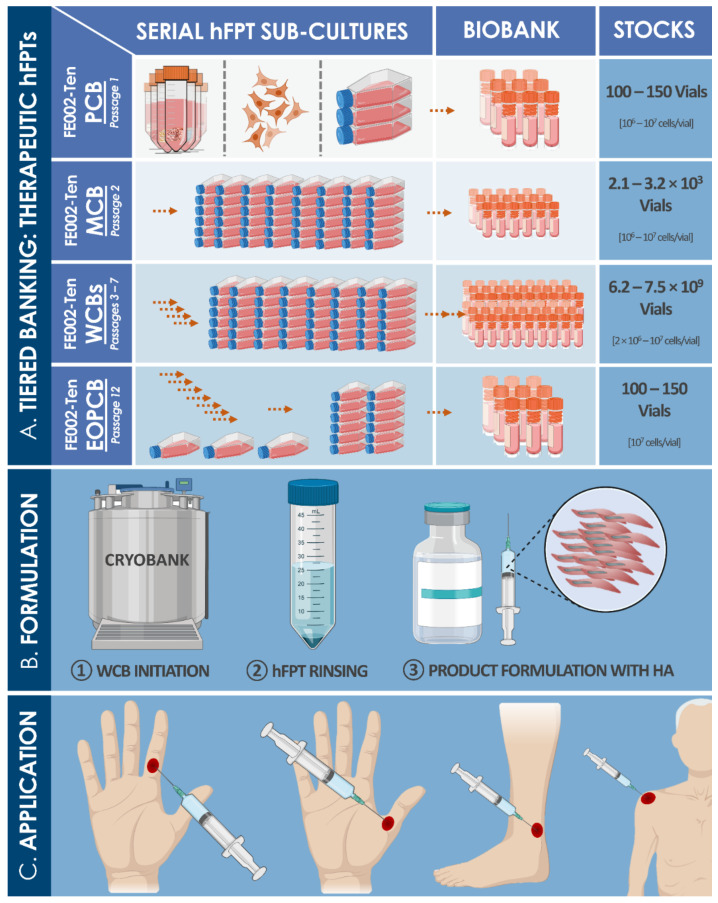
Schematic workflow representing optimized and standardized multi-tiered hFPT banking (e.g., enzymatically isolated FE002-Ten cell type) and standardized transplant preparation for tendon injury and defect treatment. (**A**) Important in vitro optimization steps enabled selection of optimally adapted FBS lots, cell culture surfaces, and culture conditions, ensuring optimal efficiency of manufacturing. Appropriate technical specifications and rigorous testing enable the manufacture of highly homogenous and stable therapeutic cellular materials to be applied in regenerative medicine (e.g., extemporaneous on-demand production of standardized transplants for hand or articulation tendons) or in biotechnological applications. Particularly, specifically devised multi-tiered hFPT biobanking workflows enable the rapid generation of therapeutic materials for the potential treatment of millions of patients. (**B**) For extemporaneous standardized transplant manufacture, hFPT WCB vials are removed from liquid nitrogen storage, cells are rinsed for cryopreservation medium removal, and further formulated with adapted cell delivery vehicles (e.g., HA-based hydrogels) in view of rapid (i.e., within 72 h of cell initiation) clinical delivery of viable cellular constructs. (**C**) Following standardized transplant product preparation, localized clinical administration should occur rapidly for management of defects in hand tendons, rotator cuff tendons, or Achilles’ tendons. EOPCB, end of production cell bank; FBS, fetal bovine serum; HA, hyaluronic acid; hFPT, human fetal progenitor tenocytes; MCB, master cell bank; PCB, parental cell bank; WCB, working cell bank.

For cell transplantation purposes, tendon tissue samples from the FE002 organ donation were differentially and parallelly processed under the Swiss FPC transplantation program for hFPT PCB establishment (Figures S1–S4). Thereafter, important manufacturing process optimization steps took place during the pilot cell banking phase, enabling efficient establishment of robust technical specifications and extensive material lots of MCBs, WCBs, and EOPCBs of FE002-Ten cells (Figure 2 and Figure 3). Optimal cell proliferation conditions were obtained with a Sigma^®^ FBS lot (i.e., clinical-grade) for culture medium supplementation, using 75 cm^2^ TPP^®^ cell culture flasks, cell seeding at 1.5 × 10^3^ ± 100 viable cells/cm^2^, culture medium volumes of 10 mL/flask, culture medium exchanges twice/week, and 14–15 days of culture before harvest of confluent cell monolayers (Figure 2, Table 1, results partially shown). Cell banking and tier nomenclature depended on the passage numbers rather than on cell population doubling values (PDV), since the seeding and harvest parameters were consistent throughout production passages (Figure S4).

The above-mentioned optimized culture conditions and specifications were applied for the subsequent banking steps of FE002-Ten cells. Therein, for the large-scale cell banking campaigns of hFPTs, specific average population doubling times (PDT) ranged from 82 h (i.e., Passage 1) to 111 h (i.e., Passage 12) (Table 2). Proliferative cell morphology, cell viability, and batch sterility (i.e., contamination absence) were systematically assessed throughout passages and were not found to be out of specifications (Table 2). With individual vials containing 10^6^ to 10^7^ viable FE002-Ten cells upon freezing, manufacturing lot sizes generally reached 80–250 vials when considering 80 T75 flask batches for expansions (Table 2, Figure 3). Proliferative cell morphology during expansions was consistent and fibroblastic in nature (i.e., elongated spindle-shaped cells) and characteristic for this specific cell type. During serial expansion rounds (i.e., Passages 1 to 12), relative hFPT viability upon recovery, assessed by Trypan blue staining, ranged from 97% to 100%, confirming excellent resistance of such cells to cryopreservation in a DMSO and FBS-based medium. The multi-tiered cell banking workflow for the FE002-Ten cell type described herein was sequentially repeated and validated at least five times (Figure 3). Establishment of WCB lots at Passages 3 to 7 enabled the devising of a sustainable sourcing model for therapeutic hFPTs, thereby virtually abolishing the need for repeated original organ donations (Figure 3). Specifically, the adopted model for theoretical projection of obtainable P7 vials from a single donor showed that up to 7.5 × 10^9^ vials could potentially be generated by standardized processing and adequate banking, confirming the high technical value of properly exploited hFPT cell sources (Figure 3).

### 3.2. In Vitro Soft Agar Colony Formation Assays for hFPTs

At the defined timepoint of assay ending (i.e., 12 days after initial incubation), HeLa and HCT 116 positive control cell lines had formed large (i.e., >200 µm in diameter) and non-adherent mass colonies. In contrast, FE002-Ten hFPTs were not observed to have proliferated and had formed no aggregates or colonies in all the different investigated assay settings. Specifically, suspended hFPTs conserved a round phenotype in the soft layer of the agarose gel, and hFPTs located at the interface between solid and soft layers were not observed to have adhered or to have adopted the characteristic fibroblastic phenotype usually observed in standard in vitro monolayer cultures.

### 3.3. In Ovo Standardized Toxicity Study of hFPTs

**Figure 4:**
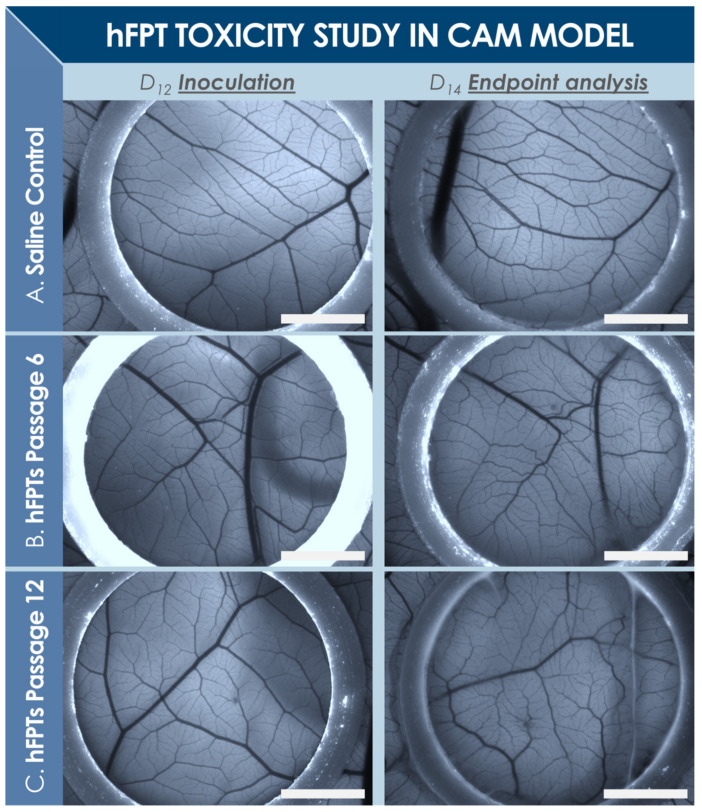
Representative data of standardized CAM assay for evaluation of hFPT toxicity in ovo. (**A**) Saline control group. (**B**) hFPT at P6 group. (**C**) hFPT at P12 group. Embryo viability and quality of angiogenesis served for endpoint assessment of test-item effects. For test-items, the quantity of cells distributed on each egg corresponded to the quantity of cells formulated and administered in the GLP rabbit safety study (i.e., 2.4 × 10^6^ cells/dose). Data are presented for eggs N°4, N°11, and N°24 for controls, P6, and P12 groups, at days 12 and 14, all in respective order. Scale bars = 3 mm. CAM, chorioallantoic membrane; GLP, good laboratory practice; hFPT, human fetal progenitor tenocytes.

The main reason for including an O-ring in the presented standardized CAM assay was to ensure a homogenous disposition of the membrane, with a flat surface ensuring good photographic image acquisition. Generally, the O-rings were not observed to have moved on the membrane, and the inoculated cell suspension was not observed to have leaked out of the area delimited by the O-rings. Experimental results indicated that hFPTs at P6 and P12 did not incur embryo mortality or abnormal formation of vasculature in the CAM model, as compared to normal saline controls (Figure 4). Briefly, upon reception of the egg batch (i.e., day 0), 60 intact white eggs were incubated. On day 12, 24 h following removal of the shell during large opening creation, 39 eggs were assessed as viable and were available for testing (Figure S5). Control, P6, and P12 groups were randomly allocated with 13 eggs per group, respectively. After incubation and at the time of analysis (i.e., day 14), all eggs were assessed as viable across the three groups. The effects of the tested cellular materials on chicken embryo viability and development were therefore considered to be non-significative after 55 h of direct contact with CAM membranes. Further macroscopic and microscopic endpoint investigations around differential angiogenesis between test groups did not reveal any signs of abnormalities or any differences across the three groups. In particular, comparative assessment of the CAM vasculature was made visually for vessels observed within the O-rings, in order to underline macroscopic and microscopic similarities and differences in eggs included in the assay between day 12 (i.e., inoculation) and day 14 (i.e., endpoint imaging, Figure 4). Specifically, observed vessel length, angles, and branching points were compared for each egg. Therein, despite observed relative internal shifts in vessel disposition within the developing membrane, the general structure of the vasculature was observed to be conserved. In particular, considered vessel lengths and branching points were highly conserved over the course of the assay. Vessel relative angles were mainly conserved for main vessels, but were modified for secondary vessels, in accordance with the development thereof. The diameter of main vessels appeared slightly reduced in the endpoint images, but this aspect was attributed to membrane growth and development, as well as slight differences in the focus settings of the stereomicroscope camera used for imaging between initial and endpoint image acquisition. Finally, the microvasculature was observed to have developed over the course of the assay, wherein the microvasculature was observed to generally be more dense, more branched, and of relatively greater length at the 14-day timepoint, in accordance with normal development of the CAM.

### 3.4. In Vivo Preliminary Safety of hFPTs in a Pilot GLP Study of Rabbit Tendon Defect

#### 3.4.1. Treatment Administration and General Animal Observations

Preparation and administration of formulated standardized transplant prototypes were assessed as satisfactory, based on hFPT cellular viability maintenance and simplicity of product handling in the specifically developed model of rabbit partial-thickness patellar tendon defect (Figures S6–S9). Formulation details and product quality controls are presented in Supplementary Materials and Figure S9, respectively. No surgical intervention-related or test-item-related mortality were observed for animals included in the study. No test-item-related clinical signs or test-item-related modifications in recorded physiological parameters were observed. No abnormality in animal body weight was observed. No lameness and no apparent abnormal clinical symptoms were observed prior to sacrifice of all study animals at 6 weeks post-intervention.

#### 3.4.2. Study of Animal Blood Compositions

No important or significant changes were observed in blood routine parameters between the time of treatment administration and sacrifice. A special focus was placed on leukocyte concentrations. A global mean concentration of (10.5 ± 2.3) × 10^3^ leukocytes/mL (*n = 6*) was present prior to intervention and exactly the same mean concentration of (10.5 ± 1.7) × 10^3^ leukocytes/mL (*n = 6*) was present 6 weeks later, prior to sacrifice. The rabbits which had received the test-items presented a mean concentration of (10.2 ± 2.2) × 10^3^ leukocytes/mL (*n = 3*) at the moment of sacrifice, while the rabbits which had received the reference items presented a mean concentration of (10.9 ± 1.3) × 10^3^ leukocytes/mL (*n = 3*). No rabbits had experienced major individual changes in leukocyte titers. Mean values are presented with corresponding standard deviations (SD).

#### 3.4.3. Macroscopic Evaluation of Harvested Tendon Tissues

Five of the six rabbits were scored as 0 on the 8-grade scale and one rabbit was scored as 1 (Table A2). The surgeon who performed the macroscopic evaluation did not notice any inflammation, tissue adhesion, fibrosis, or inconsistencies in defect filling, except for one tendon injected with the test-item, which presented mild inflammation. There was a very slight change in color noted in five out of the six considered tendons (i.e., three in the test-item group and two in the reference item group). The contralateral untreated patellar tendons all presented normal structures, without any noteworthy observations, and they all would have received a 0 score with the grading employed for macroscopic evaluation (Table A2).

#### 3.4.4. Microscopic and Histological Evaluation of Harvested Tendon Tissues

**Figure 5:**
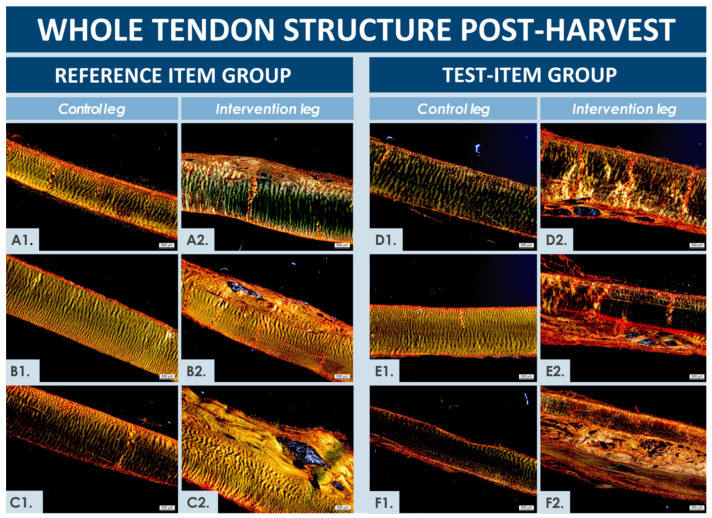
Full-thickness structure of the operated and treated or contralateral untreated rabbit patellar tendons, harvested, stained with Picrosirius Red, and observed under polarized light. (**A1**–**F1**) The contralateral untreated tendons all presented normal structures. (**A2**–**F2**) The healing process was on-going after 6 weeks for the operated and treated tendons in both groups. The artificial defect zones were recognizable due to relatively less organized collagen tissue, localized relative tissue enlargement, and presence of small cavities in some cases (**B2,C2**). Remaining biomaterial may be observed in operated tendons having received the cell therapy (**D2**–**F2**). It is to note that, based solely on the photographic data presented herein, the treated reference item group (i.e., **A2**–**C2**) shows better global structure of the harvested tendons than the treated test-item group (i.e., **D2**–**F2**). However, this aspect is to be highly relativized, based on the variability between animal test-subjects, the available and presented histological pictures, and the alternative complementary readouts used for the assessment of all operated and treated tendons. Particularly, the full integration of all investigated readouts (i.e., macroscopic and microscopic investigation, histology, variety of observed tissue zones) was in fine retained for the discussion and conclusions drawn about the safety of the proposed cell therapy in the rabbit model of interest. Scale bars = 500 µm.

**Figure 6:**
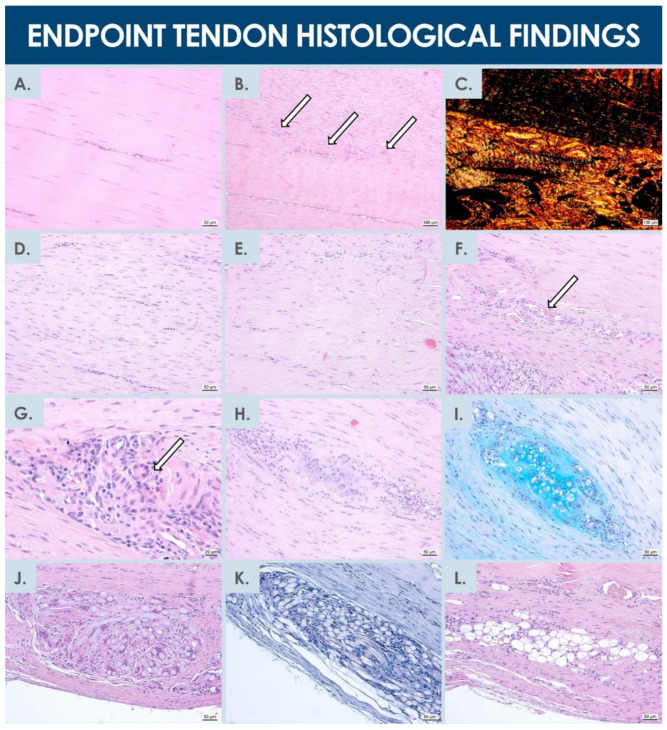
Microscopic patterns which could be observed on harvested tendon sections stained with hematoxylin–eosin (HE), Picrosirius Red (SR), or Alcian Blue stains (AB), and which were taken into account during the scoring process. (**A**) Normal structure of an untreated tendon with tenocytes dispersed in the matrix and with presence of a naturally aligned blood vessel, HE staining. (**B**) Delimitation of a defect (i.e., indicated by arrows) in an operated tendon, HE staining. The upper part of the picture shows the repaired defect and the bottom part shows normal tissue structure with a standard blood vessel. (**C**) The delimitation of a defect could be facilitated by an observation with Picrosirius Red staining under polarized light. The upper part of the picture shows a natural structure with good alignment and low diffraction of light, while the bottom part shows more light reflection due to misalignment of collagen in the matrix. (**D**) Hypercellularity, HE staining. (**E**) Disorganization of tendon structure, with immature or disrupted tendon structure, with undulating fibers, HE staining. (**F**) Hypervascularity and presence of transverse vessels (i.e., indicated by the arrow), HE staining. (**G**) Immune cell reaction and presence of a foreign body giant cell (i.e., indicated by the arrow), HE staining. (**H–I**) Presence of remaining biomaterial, detectable with HE staining. Alcian Blue staining allows for an easier detection of remaining biomaterial, with bright blue coloring. (**J–K**) Presence of suture material, recognizable with HE staining. The AB staining allows to exclude the presence of biomaterial, as there is no bright blue coloring. (**L**) Presence of open spaces, HE staining. Scale bars = 20, 50, or 100 µm.

Similar to macroscopic assessment results, the contralateral untreated patellar tendons all presented normal microscopic structures, without any noteworthy observations. The operated and treated tendons (i.e., using test and reference items) were all in the process of healing, as would be expected 6 weeks after the intervention, and were observed to be thicker than their respective contralateral untreated tendons (Figure 5). Specific and individual remarkable structures could be observed in normal and operated tendons at the 6-week timepoint after the operation (Figure 6). Gross evaluation allowed to localize the artificial defects and to estimate size and apparent quality of repair thereof. The defects extended from one-third to two-thirds of the tendon in depth, wherein the bottom edges were detectable in all tendons. The distal edge of the defect was visible in only one tendon (i.e., test-item group) and the proximal edge was visible in two tendons (i.e., test and reference item groups), but were diffused in all other considered tendons. Distinction between surgically created flaps and repair tissue was arduous. Surface tissues were mildly reactive for all tendons, except for one sample in the reference item group, where the tissue was highly reactive. The suture material was not fully resorbed 6 weeks after operations and was discernible in four tendons (i.e., two samples in each group). Inflammatory cells were present around suture material remnants, as would be expected. Residual biomaterials (i.e., hydrogel) could be macroscopically detected in one tendon sample from each group and in a third tendon sample from the reference item group during microscopic evaluation. Considered areas were relatively larger in the tendons from the test-item group and were more densely populated by cells. One tendon from each group presented small cavities close to the upper border, probably in the area of the surgical flap. One tendon injected with the test-item presented small cavities in the midsubstance (Figure 5 and Figure 6).

Hypercellularity and hypervascularity were visible in all the repaired regions, as would be expected in tendon tissue undergoing active regeneration (Figure 5 and Figure 6). The tissue fibers were generally oriented longitudinally, although not perfectly aligned and comprising zones of higher disorganization in less mature repair areas (Figure 5). Necrosis was not observed, and no signs of immune reaction were noted, except around foreign suture materials, which is to be expected for resorbable sutures at this timepoint. In addition, calcification (i.e., identified by von Kossa staining) was never observed (data not shown). The higher magnification microscopic evaluations enabled to allocate nominal scores to the repaired tissues (Figures S10–S12, Table 3 and Table A3). Results of both considered zones were averaged for each rabbit and were presented as individual nominal scores in the condensed contingency table for the three rabbits within each group (Table 3). High hypercellularity was observed in all evaluated zones, except in one zone from the reference item group, where it was observed as moderate. There were signs of hypervascularization in all the evaluated zones, except for one tendon in the test-item group. Macrophages were noticeable in the repaired tissues, but groups of foreign body cells, lymphocytes, or granulocytes were never evidenced in the observed zones (i.e., although presence of foreign body cells was visible around suture materials, not included in the evaluation zones). The presence of remaining biomaterials was noted in one tendon from the test-item group, where it was found to be large, clustered, and highly cellular.

Such presence was also noted in two tendons from the reference item group, but in relatively smaller areas, with more scattered patterns between fibers and with hypercellularity. Although not specifically studied and non-significantly different, a trend was observed in the cell therapy group, wherein inflammation around non-resorbed sutures was relatively less important than in the reference item group. The repaired ECM was tendon-like in two tendons from each group and appeared pre-differentiated and immature in one tendon from each group. The directionality was evident in five tendons, although slightly misaligned as compared to normal tendon tissue. In the last tendon from the test-item group, the fiber directionality was not discernible in the focused zones (Figure 5F2). Basing significance on a *p* value below 0.05, Fisher’s Test for exact count data, applied to analyze the individual parameters and study groups using results of microscopic scoring, showed no dependence between individually assessed parameters and the study groups (Table 3). Finally, based on ALU staining results, no remaining human cells were detected in all the processed materials after the 6-week pilot safety study, and no related or remaining abnormalities were observed.

## 4. Discussion

### 4.1. Optimized Multi-Tiered hFPT Banking for Safe and Sustainable Tendon Cell Therapies

Within pragmatic development of cell-based product manufacturing workflows at industrial scales for product registration and eventual commercialization, several aspects such as effectiveness and incurred costs of cell banking play major roles. Therein, basic parameters such as those investigated herein for technical specification optimization (e.g., culture conditions, culture maintenance workflows, contact-process consumables, and raw materials) are prerequisites for sound technology transposition (Figure 2 and Figure 3, Table 1). Specifically, highest attention must be paid to the kind of plastic surfaces used in cell expansion vessels, including the surface area of individual vessels, and to the exact source of medium supplementation (i.e., lot number for clinical-grade FBS). Over 30 years of experience by our group in cell banking technology transfers and transposition have outlined that, without proper benchmarking of both culture vessels and serum sources for each new cell type, manufacturing yields may be halved as compared to optimized conditions, incurring considerable additional costs.

As we have previously shown, hFPTs are highly attractive candidates for tendon tissue engineering applications from a technical and quality point of view, as they are highly stable with regard to tenogenic properties in culture, are tissue-specific, and expand rapidly (Table S1, Figure S13) [49]. Additionally, such cellular active substances may be effectively delivered in appropriate hydrogel formulations for localized stimulation of tendon healing in partial-thickness wounds [48]. Relatively increased ECM production and deposition is achieved by using hFPTs in appropriate therapeutic conditions, as compared to primary adult tenocytes [48,49]. Cultured sources of tenocyte progenitors are similar to stem cells albeit being relatively restricted in terms of potency. Despite their pre-terminal differentiation state, these cell populations exhibit a lack of specific tendon markers, which complexifies simple in vitro characterization and prompts the use of marker panels (e.g., type I collagen, scleraxis, and tenomodulin) for identity or purity assessments [50,51,52,53,54,55]. As for other primary progenitor cell types, hFPTs adapt well to extensive tiered biobanking frameworks, constituting stable and sustainable sources of high-value therapeutic materials to be converted into tissue engineering products (e.g., cell-seeded biocompatible scaffolds) [49]. Original cell banking optimization work presented herein has stressed the need for extensive and specific validation of each aspect of cell lot manufacturing, for insurance of optimal quality of released batches (Table 1 and Table 2). Alternative development of injectable formulations (i.e., using medical device vehicle scaffolds) destined to stimulate tissue regeneration (e.g., localized injuries, small fissures, or partial ruptures) may be of high-value for effective management of hand tendon injuries, without the need for extensive cell-seeded construct preculture periods [37,48].

Overall, cultured hFPTs such as FE002-Ten cells are characterized by optimal stability, consistency, and traceability, which are prerequisites for all starting materials destined for allogeneic cell therapy applications [49,65]. Proposed bioprocessing methodologies and subsequent multi-tiered cell banking for clinical-grade progenitor cell sources (e.g., FE002-Ten cell type) are primordial for quality assurance and sustainability of the material source. Therein, when fully exploiting technical capacities for large manufacturing yield obtention in GMP settings, adapted transposition eventually results in minimal direct costs of therapeutic raw material production. Appropriate product release and characterization testing (i.e., in GMP settings) of hFPT MCB and WCB lots may comprise recovery assays, isoenzyme and sterility testing, specific testing for endotoxins, mycoplasma, viral contaminants (e.g., adenovirus, B19 parvovirus, BPyV, EBV, HPV, HBoV, HAV, HBV, HCV, hCMV, HIV, HTLV, HHV, KIPyV, orthomyxovirus, paramyxovirus, picornavirus, reovirus, West Nile virus, WUPyV, SV40), reverse transcriptase activity testing, or quantitative transmission electron microscopy (TEM) of cell sections for the detection of pathogenic contaminants (e.g., viruses, virus-like particles, mycoplasma, yeasts, fungi, bacteria) [61]. Such standard industrial testing, routinely included in related technical specifications, may be adapted to workflows for large-scale production of therapeutic hFPTs. This is possible due to extensive banking potentials, cell type consistency, and cell stability over defined in vitro lifespans (e.g., stable evolution of FE002-Ten karyotype, potency, and surface markers over passages) [49].

### 4.2. hFPTs for Formulation of Standardized Transplants in Tendon Tissue Engineering

In the present work, pilot product formulations for hFPT delivery were selected and adapted based on the requirements of the considered tendon wound types, wherein the chosen CAM and rabbit models were parallelly optimized for the preliminary toxicity and GLP safety studies, respectively. A rabbit model was chosen for the pilot in vivo safety study, for consistency in view of further efficacy investigations, based on the similarities between human and rabbit tendons (e.g., size and structure, sequential healing phases) and based on the fact that it is the most frequently used GLP animal model for tendon engineering research. Notwithstanding specific and respective anatomical junctions and structures, rabbit flexor tendons resemble human tissues in diameter and in the presence of a synovial sheath. Such characteristics are different from tendons of mice and rats (i.e., structure, size, relative load bearing, availability of sufficient tissue for in vivo imaging and eventual mechanical testing) and provide an acceptable compromise in terms of costs and model relevance, as compared to more challenging large animal models. The patellar tendon model was chosen for intervention due to optimal accessibility and relative dimensions in view of surgical defect creation and product administration. A partial thickness tissue defect model was retained, to limit the risk of complete structural failure, which was furthermore mitigated by suturing the tissue after product delivery. Following surgery, total functional recovery was therein restored in all animals after several hours, as they all managed to bear weight and ambulate on their respective hindlimbs. Despite inherent advantages of rabbit patellar tendons for defect model elaboration, effective defect volumes remain limited and prompt the development of highly concentrated products. Preliminary cadaveric studies had shown that the maximal volume of injection that would fit in the created defects would approximate 25–40 µL (Figures S7 and S8). Therefore, concentrated formulations were prepared, to inject at least 2 × 10^6^ hFPTs into the limited volume of the defect. In such a high concentration, the cells represented approximately one-third of the final preparation volume. Therein, cells were diluted in a minimal amount of PBS to facilitate manipulations, while maintaining a final concentration of 50% HA in the formulation and thereby conserve sufficient viscosity to prevent formulation leakage from the defect (Supplementary Materials, Figure S8).

For appropriate comparison to the test-items, the reference items contained the same HA proportion, to exclude a potential differential intrinsic therapeutic effect thereof. Inclusion of a third experimental group, operated for defect creation but not treated with either items would be of interest to assess the overall effects of both hFPTs and of the hydrogel component of the formulation versus natural tissue healing. With regard to the test-items, a relatively higher viscosity was noted, due to the presence of hFPTs in high concentration. Furthermore, due to the relatively small scale of product preparation and excess fabrication for process validation and cell viability testing, low manufacturing yields characterized the preparation of test-items, wherein only 150 µL of dispensable formulation were obtained after processing of 350 µL bulk product through application devices (i.e., syringes, needles, catheter). Taking into account additional margins of safety for assurance of standard doses, approximately 2.4 × 10^6^ cells were injected in the tissue defects, as compared to the 28 × 10^6^ cells used to prepare one syringe, prompting the further need for process optimization after appropriate validation phases. Conversely, when considering larger defects or patients requiring larger product volumes, such scale-related problematics may be partially mitigated. It is projected that for human applications, a concentration of (2.25 ± 0.25) × 10^6^ hFPTs in a volume of 500 µL of product would be adequate. Such proportions were previously experimentally validated, with 60–70% dispensable cell quantities [48]. In the present experimental setting, the quantity of cells dispensed in the tendon defects was inferior to 3 million on the 28 million cells originally loaded with the syringe. However, we would not qualify the cells remaining in the syringes after administration as lost, as the syringes were kept and further studied in validation assays (i.e., cell viability, cell proliferation studies, and general observation of the pharmaceutical form, see Supplementary Materials). Therefore, although a significant volume of suspension was lost in void volumes and several million cells were not used, a majority of formulated hFPTs were used either in the in vivo study, or in related in vitro testing of the product dosage form. Within the development of a final product, the cell viability validation phase would not be performed at each administration, but appropriate quality attributes would rather be standardized before a final product design freeze, as well as the stability of the preparation. Therefore, the hydrogel volume and cell quantity per syringe may be modulated for optimized efficiency, minimizing the number of cells which do not reach the administration site. In any case, a cell fabrication excess is necessary due to void volumes in needles/catheters during administration, but an optimized final formula for human use could eventually be proposed, wherein the majority of therapeutic hFPTs would be injected in the patient. Manufacturing efficiency could furthermore potentially benefit from pooled bulk product processing for final individual product preparation, with final individual conditioning in single-dose syringes. Results have outlined the importance of an adequate cell delivery vehicle in order to avoid heterogeneity and persistence of biomaterial formulations in the treated defects (Figure 6).

### 4.3. High Clinical Need for Effective and Efficient Biological Products for Tendon Repair

Tendinous tissue affections (i.e., acute or chronic) represent major challenges in modern reconstructive surgery, with highly variable outcomes and rare optimal healing evolution (Table S2) [18,19,22]. Acute extensor and flexor tendons, rotator cuff tendons, and hand tendons are most exposed to accidental trauma or overuse-related premature wear [4,67]. When considering particularly exposed structures (e.g., hand tendons), injuries may be classified as simple acute lacerations or complex tendon laceration with associated trauma to surrounding tissues. Therein, specific treatment modalities and prognosis highly depend on the location and extent of the damage. In most clinical cases of simple acute laceration, tendon primary suture and a zone-specific rehabilitation program may be sufficient to restore adequate strength and function. However, in everyday clinical practice, patients are generally only allowed to apply full strength and load after 10 to 12 weeks onto a limb presenting a tendon injury [4,9,11,15]. Consequently, the overall cost of such injuries is elevated, even for fully observant patients who diligently follow the established rehabilitation programs, motivated by an increased desire for total functional recovery [15,16].

Tendon healing processes are lengthy, as after the initial inflammatory response, the proliferative or reparative phase begins in a delay of 4 to 7 days [68,69]. Thereafter, a remodeling phase starts 6 to 8 weeks after the injury, accompanied by the development of a more structured architecture. Therein, fibrovascular tissue activity decreases and collagen fibril structure is remodeled with a more linear orientation [70]. In the renewed tendon, the fiber pattern is thereafter more disorganized, with tendon fibers randomly oriented and high proportions of linking, resulting in scar tissue formation, reduced elasticity and mobility, with an increased predisposition for re-rupture [23,71]. In the case of extensive injuries with volumetric tissue defects, the use of tendon grafts is almost always mandatory. Additionally, tendon grafting may be required to repair an injury to a flexor tendon, even if there is no tissue defect. Autologous palmaris and plantaris longus tendon grafts are the first options, yet sometimes the segmental loss exceeds the available donor tissue. Moreover, successful treatment of a tendon injury and time to restore adequate joint mobility rely on the effective prevention of tendon adhesion formation, which remains as a basic unresolved complication in tendon surgery [72].

High clinical need for fast and effective tendon tissue healing promotion strategies, as well as the need for sufficient donor tissues, are well established. This availability gap explains why tissue engineering and regenerative medicine started to focus on developing new therapeutic tools, as well as new artificial tendon substitutes. Indeed, tendon grafting is commonly required to repair an injury to a flexor tendon. However, there exists a lack of suitable graft material, and controversy remains as to which materials are best suited for flexor tendon repair (i.e., extrasynovial versus intrasynovial flexor tendons). Efficacy of tendon transfer procedures might furthermore be hindered by high rates of graft degeneration and could largely benefit from stimulation by therapeutic cellular components, to ensure necessary improvements in biomechanical properties [25,26]. Bioengineered therapeutic constructs of diverse origins have been proposed to supplement classic therapeutic management of tendon injuries. Considered scaffolds have comprised decellularized tendon matrixes (i.e., human cadaveric or equine origin) or artificial equivalents, tailored to ensure biocompatibility and acceptable ranges of mechanical properties. The aim of using such materials is to approach the efficacy outcomes and functional parameters of autologous vestigial tendon transfers [29,30,31,32,33,34,35,36]. Despite large acquired experience and hindsight, it appears that pure surgical management of tendinous affections remains unsatisfactory for appropriate healing. Therefore, applied and translational research has been prompting the development of additional or complementary therapeutic solutions such as cell-based therapies [8,9,17].

### 4.4. Limited Available Cell Therapy Options for Tendon Regeneration

In the tendon transplantation context, devitalized allografts have been used in mouse models for flexor digitorum longus repair, however they did not demonstrate improved structural and mechanical properties or less formation of adhesions when compared to the use of autografts [72]. Considering alternative regenerative medicine approaches, numerous cell types and sources have been proposed for tendon regenerative medicine applications, comprising adult tenocytes, tendon sheath fibroblasts, epitenon tenocytes, specific stem cells (i.e., bone marrow or adipose-derived), placenta cells, amniotic cells, fetal progenitor cells, and platelet-derivatives [37,39,40,42,47,73]. Mesenchymal stem cells (MSC) were used on acellularized allogeneic tendons to fill a profundus tendon defect in rabbits with good viability rates at 6 weeks [74]. In another rabbit model, bone marrow-derived MSCs were implanted into lacerated and sutured Achilles’ tendons, ameliorating the biomechanical and histological properties in the early stages of tendon healing [43]. A study in a rat model suggested that bone marrow-derived MSCs, administered in a fibrin glue carrier, may improve rotator cuff tendon healing and reduce the incidence of re-tears as compared to controls [75]. As carriers of MSCs, various kinds of bio-scaffolds have been developed. Some have shown improvement in force to failure, better tensile stiffness, and no ectopic calcification when used to repair tendons in rabbits [76,77].

Another group achieved to successfully develop a xenograft model from embryonic stem cells (ESC). The human ESCs were differentiated to MSCs and then introduced into a fibrin scaffold. Then, the whole construct was grafted in a rat patellar tendon. This construct remained viable for a minimum of 4 weeks and activated the endogenous tendon regeneration process without the formation of ectopic tissue or teratomas. Moreover, such constructs attained better biomechanical properties than controls. However, the presence of calcification in some of the cases forced the authors to conclude that there is the need to develop better differentiation techniques for the use of ESCs as the primer cell type of tendon tissue engineering [40]. Furthermore, a blinded placebo-controlled large animal study (i.e., equine model) over 8 weeks revealed substantial and clinically relevant improvement in the healing of tendon injuries after intra-lesional injection of allogeneic fetal-derived ESCs. The MRI and ultrasound analysis of the treated tendons revealed important improvement in the tissue architecture, with a more linear pattern of the tendon fibers when compared to controls [78].

In human clinical investigations, a study on 14 patients with rotator cuff defects reported treatment with autologous mononuclear stem cells extracted from the iliac crest. The cells were then injected into the borders of the tendon after conventional surgical repair. According to the authors, this resulted in positive findings related to the safety of the treatment and its potential to enhance endogenous tendon wound healing properties [79]. Currently, great efforts are made toward developing specific biocompatible scaffolds, wherein human natural tendon is considered as an ideal graft material. Acellularized tendons can be reseeded with MSCs and then used to tentatively promote faster graft incorporation and in situ remodeling. This would permit early motion in order to prevent adhesion formation [80]. Alternatively, platelet-rich plasma (PRP) has been investigated in many large animal preclinical and human clinical settings for enhancing rates of tendon repair [81,82,83]. Studies on the effects of PRP in rotator cuff surgery showed promising results, with increased functional outcomes assorted to reduced post-operative pain [84,85]. However, similar studies in the repair of Achilles tendon tears or in chronic lateral epicondylitis provided mixed results [86,87,88,89,90]. Furthermore, other kinds of cell-based therapies have been successfully used for the treatment of lateral epicondylitis in recent years. Collagen-producing cells derived from dermal fibroblasts were developed and administered to 12 patients with lateral epicondylitis in a specific study. The Patient-Rated Tennis Elbow Evaluation score was measured over a period of 6 months at regular intervals, along with ultrasound measurements. The median score decreased significantly at 6 months, with 11 out of 12 patients reporting satisfactory results [44].

Overall, the contrasted results of preclinical and clinical investigations around the use of various proposed therapeutic cell sources (e.g., ESCs, MSCs, PRP) have outlined the relative complexity of tendon tissue treatment with regenerative medicine approaches. In addition to the tissue-specific requirements and the need for tailored cell-delivery vehicles, inherent limitations of considered cell sources have comprised poor homogeneity, high technical requirements in culture, and inconsistent effects on tissue repair or regeneration. Therefore, research was focused on tissue and cell sourcing for sustainable provision of stable, safe, and consistent biological raw materials, such as appropriately isolated cultured primary hFPTs [49]. Such considerations may represent a key element for successful delivery of active cellular components to highly dense tissues such as tendons.

### 4.5. FE002-Ten hFPTs as Safe Cell Sources for Tendon Tissue Engineering

The main safety-oriented goal of the present study was to preliminarily assess specific hFPTs, which were investigated in soft agar transformation assays, CAM assays, and in the rabbit GLP pilot safety study (Figure 4, Figure 5 and Figure 6, Table 3) [91,92]. Therein, absence of tumorigenic potential was demonstrated in vitro, with the absence of hFPT ability to grow independently from adherent settings, contrasting with the behavior of characterized cancerous cell lines. Such results were in line with previous in vitro investigations on potential tumorigenicity of the considered cell type (i.e., FE002-Ten cells), which had been preliminarily excluded based on the fact that they were found to be incapable of anchorage-independent growth when extensively incubated in HA-based hydrogels (results not shown) [48]. Furthermore, absence of acute toxicity was confirmed, as clinically relevant hFPT doses did not promote chicken embryo death or occurrence of abnormalities in CAM angiogenesis. Additionally, in vivo safety was preliminarily shown, as products did not cause observable local immunogenic or tumorigenic effects in the animals receiving the test-item compared to those receiving the cell-free reference item. Within the in vivo GLP preliminary safety study presented herein, creation of tissue defects and hydrogel implantation within the midsubstance of tendons was intended to evaluate potential biological reactions to the implanted products during preliminary stages of the repair process. Therein, the size of rabbit patellar tendons was found to be sufficient for delivery of relevant cell doses [93]. As expected, the contralateral untreated rabbit tendons all presented a normal structure 6 weeks post-intervention, without specific observations to report (Figure 5). Operated tendons presented many similarities between treatment groups, wherein the applied Fisher’s exact test did not detect any significant dependency between individual parameters and study groups (Table 3). Tissues from both treatment groups were at similar stages of healing upon examination.

The general healing process in tendon tissues is clearly described in the literature and relies on three main phases, with an initial inflammatory stage occurring rapidly after trauma. Therein, multiple cell types infiltrate the wound, particularly erythrocytes and inflammatory cells, and numerous released factors initiate angiogenesis and tenocyte attraction. During the proliferative stage (i.e., second step), the repair site is highly cellular, with formation of an extensive blood vessel network and ECM deposition under tenocyte control. Macrophages are still present but in lower amounts and assist in the repair process. Thereafter, during the remodeling stage, cellularity decreases, and tissues organize longitudinally, with an evolution from cellular to fibrous typology, with eventual creation of scar tissue in adults [20,23,24,25,26,94,95,96]. Based on these specificities, it was estimated that considered operated rabbit patellar tendons were between the proliferative and the remodeling phase. Indeed, hypercellularity and increased vascularization were visible in both treatment groups. Vascularity was increased both in the frequency and size of blood vessels, with the presence of transverse vessels (i.e., absent in normal tissues). Furthermore, indications of remodeling stage initiation were noted, as the matrix appeared tendon-like in two thirds of considered tissues and fiber directionality could be observed in five out of the six tendon samples. Supplementary evaluations could potentially enable to outline differences between both treatment groups, as the appearance of scar tissue takes place in the final stages of healing. Therefore, later timepoints than the one selected herein (i.e., 6 weeks post-intervention) would yield important information regarding quality and long-term functionality of treated tendon tissues, in a controlled efficacy-oriented study of hFPT treatment [95].

Generally, the present study further confirmed the preliminary safety of hFPTs manufactured under defined specifications. Therefore, due to high robustness and versatility of such enzymatically isolated cell sources, therapeutic valorization thereof may be applied in diversified cell-based tendon repair approaches. Further optimization of both product formulation and surgical treatment procedures shall enable experimental design optimization for long-term evaluation of hFPT safety and efficacy, in attempting to achieve functional tendon tissue regeneration in preclinical and clinical settings. Furthermore, the rabbit GLP study was designed to evaluate if hFPTs were well tolerated when transferred into a host in a xenogeneic setting. Precedent studies by our group and over two decades of clinical experience around fetal dermal progenitor fibroblasts (e.g., FE002-SK2 cell type) for treatment of burn wounds and chronic ulcers in Lausanne had shown an absence of elicited inflammation and even reduced inflammatory states following repeated topical cell applications [97,98,99,100]. Based on such specific hindsight, investigations around the safety of hFPTs originally isolated from the same organ donation (i.e., FE002, 2009) were oriented toward specific endpoints, such as early tolerance of xenogeneic materials and overall effects on early tissue remodeling. This approach partly explains the limited number of rabbit test subjects and the relatively early 6-week timepoint for patellar tendon tissue harvest and analysis presented herein, while efficacy considerations were set aside for further GLP preclinical studies. Experimental observations concerning inflammation in rabbit tendons highlighted the presence of macrophages within the operated tissues, yet these are integral to the normal healing process, as described previously. Conversely, presence of giant foreign body cells, leukocytes, or granulocytes was not detected, except around suture materials which had not been absorbed before animal sacrifice. Blood samples did not indicate elevation of leukocyte titers. Overall, results have shown good safety and tolerance toward hFPTs within rabbit models and are preliminarily promising. It is nevertheless important to keep in mind the differences in immunological systems between animals and humans. Therein, further preclinical evaluation of hFPTs in alternative species would potentially allow to increase the safety profile of specific cell sources (e.g., FE002-Ten cell type) before a potential human clinical application.

### 4.6. Study Limitations and Future Directions

The scope of the present work comprised repeated experimental validation of industrial multi-tiered hFPT cell banking workflows, in vitro hFPT tumorigenicity testing, in ovo evaluation of hFPT embryotoxicity or angiotoxicity, and in vivo preliminary evaluation of hFPT safety in a rabbit model. Taken individually and considered from an efficacy point of view, the GLP pilot study did not show statistically significant improvement in the hFPT group. However, obtained results were most interesting from a safety standpoint, as no observable incurred adverse events or product rejections were noted in either group. In the present experimental setting, the absence of detectable differences at the specified timepoint (i.e., 6 weeks post-implantation) may be considered as an advantage of prime importance, demonstrating the absence of incurred early adverse effects or reactions. Furthermore, in the setting of a preliminary safety evaluation, the termination of the GLP study at 6 weeks and the restricted available tendon defect volumes were indeed not adapted to allow satisfactory evaluation of an efficacy outcome. The next phases of product effect or efficacy study shall be specifically further investigated in long-term preclinical (e.g., ovine models) and/or clinical works. Therein, evaluation of the overall outcomes of hFPT-based cell therapies will only be possible at that time. It’s important to note that based on the present results, we do not know how long the allogeneic cells (or the hydrogel) remained in place after implantation, but no human cells were detected after 6 weeks. Therefore, a larger animal model may be more suitable for specific efficacy studies, as it would also address the challenges incurred by the small size of the rabbit patellar tendon model (i.e., restricted test-item retention and efficacy assessment).

Taken individually, the presented animal study comprised a relatively low number of test subjects, due to the reasons detailed above (i.e., large clinical experience with similar progenitor cell sources) and the availability of CAM assay data to support in vivo experimentation, which was kept at a minimum by design. The relatively small patellar tendon defects presented minimal parallel inflammation, mainly around the suture materials and suspected hydrogel biomaterial remnants, identified at the time of tissue harvest. Overall, these observations would tend to favor the use of an alternate defect creation protocol (e.g., enzymatic treatment) and relative restriction in the dispensed hydrogel quantities. On a mechanistic and pharmacokinetic note, the fate of hFPTs within hosts is not yet known. Sections performed for in situ hybridization did not reveal the presence of human DNA ALU repeats at the time of analysis, as mentioned hereabove. Furthermore, tracking of human cells throughout harvested organs would confirm or infer their migration within the host. Recovery testing and viability analyses performed on test-item syringes had confirmed maintenance of hFPT viability, as well as attachment and proliferation capacities after injection (Figure S9). However, the survival kinetics of cells in the defect area post-operatively remain uncharacterized. Nevertheless, the similarity in tissue response seen in the reference item group and in the test-item group points to the lack of abnormalities and absence of notable safety concerns.

The demonstration of at least one important advantage pertaining to the efficacy of the proposed cell therapy is necessary to build the medical rationale and justify its further use in clinical trials or commercial product development. However, as mentioned hereinabove, the choice was made to limit the scope of the present work to assessment of the safety of the cell type of interest (i.e., FE002-Ten cell type), with compartmentalization of the efficacy aspect studies in the next phase of the work. Concerning the present research, the aim was to demonstrate safety in vitro, in ovo, and in vivo, which were all experimentally investigated herein and confirmed. These results are considered to be highly encouraging, as they represent a sine qua non parameter or critical quality attribute of the proposed standardized transplant formulation. The next step, currently in preparation, will be to propose a characterized final cell therapy product formulation to be tested for its efficacy in a large-scale preclinical/clinical study in view of eventual human application. Overall, considering the amount of work and the resources needed to perform each individual step, the cautious and step-by-step approach adopted herein for translational development of a tendon standardized transplant product has been an imperative within project management strategy.

Further investigations around the effects and efficacy of the considered hFPTs may comprise finer analysis of the in ovo effects of the cells on vasculature formation, for standardized quantification thereof. Biomechanical testing of the harvested tendons, as well as dimensional analysis thereof (e.g., cross-sectional area measurement) are key study parameters to be included in the further steps of efficacy characterization in large-scale preclinical studies. Such functional endpoints are critical in the devising of an efficacy model, studied for therapy rationale building and justification of further preclinical or clinical research. Therein, the selection of adequate timepoints is of equal importance, as we have shown in our parallel clinical work on cutaneous tissue reconstruction or regeneration. Therein, two decades of human application and clinical hindsight around therapeutic dermal FPCs have shown that major functional gains or benefits may only be observed in the medium or long-term after initial applications of therapeutic cells. Indeed, when considering burns, and aside from specific cases of accelerated wound closure, the main benefits evidenced for patients have been the reduced need for complication management interventions, reduced hypertrophic scarring, and overall tangible esthetic and functional gains [98,101,102]. Based on these observations, gathered over 20 years of clinical practice in our Burn Center, we are highly interested in appropriately observing the effects of specific tendon-sourced FPCs on human tendon healing, as we postulate by extrapolation that measurable benefits may be gained over longer periods, even despite the probable rapid disappearance of the initially administered therapeutic cells.

## 5. Conclusions

The present study describes industrial transposition of hFPT cell banking and preliminary safety assessments for eventual standardized transplant development, valorizing a single fetal organ donation, for envisioned optimized tendon defect repair stimulation. Therein, specific focus was set on preclinical safety of a standardized therapeutic hFPT source (i.e., FE002-Ten cell type). An optimized and validated cell banking workflow was proposed for enzymatically isolated FE002-Ten hFPTs, potentially enabling the consistent manufacture of several million therapeutic products after transposition to GMP settings. FE002-Ten cells were found to be incapable of anchorage-independent growth in soft agar transformation assays, demonstrating an absence of tumorigenic potential in defined in vitro settings. Furthermore, clinically relevant hFPT doses were characterized in ovo as non-angiotoxic and non-embryotoxic in an adapted CAM model. Lastly, an injectable hyaluronan-based hydrogel formulation yielding viable hFPTs was applied in a 6-week GLP pilot safety evaluation in a rabbit model of partial-thickness patellar tendon defect. Overall, preliminary safety of hFPT transplantation was confirmed in a xenogeneic setting, with an absence of observable immune reaction to the implanted cellular materials or acute rejection thereof. Further optimization work covering product formulation processes and delivery techniques will enable optimal efficiency of cell therapy workflows, for potentially effective tendon tissue repair promotion or regeneration. Complementing our previous reports on similar cell sources, the present study confirmed the applicability of hFPTs for large-scale manufacture and study in preclinical models, based on the safety, stability, and sustainability of such therapeutic material candidates within development of off-the-freezer regenerative medicine products (e.g., standardized transplants) and protocols. The preliminary safety data presented herein favor further investigation of hFPTs in preclinical and clinical settings, with the development of cell delivery techniques exempt of remanent biomaterial-based cell carriers. Conclusions presented in this work additionally strengthen the approach and rationale adopted for the Swiss FPC transplantation program, which continues to enable progress in novel musculoskeletal medicine frameworks directed at global betterment of patient health.

## Figures and Tables

**Table 1:** Results of multi-parameter optimization and grading of culture conditions or specifications for large-scale manufacturing of hFPT cell bank lots. Optimal culture vessels were selected based on hFPT behavior in culture and endpoint cell yields. TPP^®^ surfaces were chosen over Nunc^™^ surfaces due to supply chain issues. Optimal FBS sources were chosen based on endpoint cell yields and availability of large commercial lots. Optimal cell seeding densities, culture medium volumes, and culture periods were selected based on hFPT behavior in culture, endpoint cell yields, and cost-to-benefit ratios (i.e., sparing use of cell seed stocks, consumables, and manufacturing suite use). (−) = unsatisfactory, (+) = sub-optimal, (++) = satisfactory, (+++) = optimal. FBS, fetal bovine serum; hFPT, human fetal progenitor tenocytes.

Test-Parameter	Variables and Grading						
Culture vessel brand and surface (cm^2^) ^1^	Nunc™ EasYFlask™		TPP^®^ VENT Flasks		Corning^®^ TC Treated Flasks		
	++		++		++		
	T75	T225	T75	T150		T75	T175
	+++	+	+++	++		++	++
FBS source and lot	Sigma^®^ lot A	Sigma^®^ lot B	Sigma^®^ lot C	Invitrogen^®^ lot A		Invitrogen^®^ lot B	HyClone™ lot A
	+++	+	++	++		−	+
Cell seeding density (viable cells/cm^2^)	1.0 × 10^3^	1.5 × 10^3^	3.0 × 10^3^	6.0 × 10^3^		1.0 × 10^4^	2.0 × 10^4^
	+	+++	+++	++		+	+
Relative culture medium volume (mL/75 cm^2^)	5.0	7.5	10.0	12.5		15.0	17.5
	−	+	+++	+++		++	+
Culture period from seeding to harvest (days)	10	12	14	16		18	20
	−	+	+++	+++		+	−

^1^ Overall grades were attributed to culture surface brands based on evaluation of multiple sizes of flasks within each brand for in vitro culture of hFPTs in determined experimental conditions.

**Table 2:** Results of large-scale banking campaigns carried out for the FE002-Ten cell type following specifically optimized technical specifications and using optimized culture conditions. EOPCB, end of production cell bank; MCB, master cell bank; PCB, parental cell bank; PDT, population doubling time; WCB, working cell bank.

Passage Number	Cell Bank Tier	Average Vial Quantities/Batch at Each Harvest	PDT (h)	Viability ^1^ (%)	Sterility (+ or −)	Mycoplasma (+ or −)	Morphology ^2^ (+ or −)
1	[PCB]	80–100	82 ± 3	99 ± 1	+	−	+
2	[MCB]	80–100	82 ± 5	98 ± 2	+	−	+
3	[WCB_T1_] ^3^	200–250	84 ± 4	98 ± 2	+	−	+
4	[WCB_T2_]	200–250	86 ± 6	99 ± 1	+	−	+
5	[WCB_T3_]	200–250	88 ± 5	97 ± 1	+	−	+
6	[WCB_T4_]	200–250	87 ± 6	98 ± 2	+	−	+
7	[WCB_T5_]	200–250	88 ± 7	99 ± 1	+	−	+
8	/	20–30	91 ± 4	97 ± 3	+	−	+
9	/	20–30	93 ± 9	98 ± 2	+	−	+
10	/	20–30	100 ± 7	97 ± 1	+	−	+
11	/	20–30	108 ± 12	97 ± 1	+	−	+
12	[EOPCB]	50–60	111 ± 11	98 ± 2	+	−	+

^1^ Viability was assessed by Trypan blue staining upon initiation of hFPT vials from liquid nitrogen storage. ^2^ Morphology of FE002-Ten cultured cells was assessed by two experienced operators and consensually assessed as acceptable (+) or not acceptable (−). ^3^ WCB lots between Passages 3 and 7 were sub-tiered into intermediate tiers (i.e., Tier 1 to Tier 5) for the different characterization and validation experiments.

**Table 3:** Summary of the microscopic evaluation and scoring results of operated, treated, and harvested patellar tendons under high magnification, presented as a condensed contingency table for the three rabbits within each group. Independency between investigated parameters and study groups was assessed by using Fisher’s Test for exact count data. Ad hoc scoring definitions are presented in Table A3.

	Cellularity	Vasculature	Inflammation	Biomaterial Presence	Tissue Quality	Alignment
Score ranges	*[A–C]*	*[A–D]*	*[A–D]*	*[A–C]*	*[A–C]*	*[A–C]*
Test-item group	3 × C	1 × A; 1 × C-D; 1 × D	1 × B; 1 × B-C; 1 × C	2 × A; 1 × B	2 × A; 1 × B	2 × B; 1 × C
Reference item group	1 × B-C; 2 × C	1 × B; 1 × B-C; 1 × C	1 × A-B; 1 × B; 1 × C	1 × A; 1 × A-B; 1 × B	2 × A; 1 × B	3 × B

## Data Availability

The data presented in this study are available on request from the corresponding author. The data are not publicly available due to legal and statutory restrictions.

## Supplementary Materials

The following are available online at https://www.mdpi.com/article/10.3390/biomedicines9040380/s1. Figure S1: Swiss fetal progenitor cell transplantation program, Figure S2: Mechanical hFPT isolation workflow, Figure S3: Enzymatic hFPT isolation workflow, Figure S4: Passage nomenclature definition for FE002-Ten hFPTs, Figure S5: hFPT in ovo preliminary toxicity evaluation, Figure S6: Tendon defect model surgical overview, Figure S7: Defect model optimization–Surgical overview, Figure S8: Defect model optimization–Flexion testing, Figure S9: hFPT survival in final HA hydrogel formulation, Figure S10: Histology slides of harvested tendons under 1.25× objective, Figure S11: Histology slides of harvested tendons under 5× objective, Figure S12: Histology slides of harvested tendons under 10× objective, Figure S13: Simultaneous differential hFPT isolation workflow, Table S1: Characterization overview of the FE002-Ten cell type, Table S2: Clinically available options for tendon treatment.

## Abbreviations

AB	Alcian blue
API	active pharmaceutical ingredient
BPyV	bovine polyomavirus
CAM	chorioallantoic membrane
CD	cluster of differentiation
CHUV	Centre hospitalier universitaire Vaudois
DMEM	Dulbecco’s modified Eagle medium
DMSO	dimethyl sulfoxide
EBV	Epstein-Barr virus
ECACC	European Collection of Authenticated Cell Cultures
ECM	extracellular matrix
EDTA	ethylenediaminetetraacetic acid
EOPCB	end of production cell bank
ESC	embryonic stem cell
ESWT	extracorporeal shock wave therapy
FBS	fetal bovine serum
FIRDI	Food Industry Research and Development Institute
FPC	fetal progenitor cells
GLP	good laboratory practices
GMP	good manufacturing practices
HA	hyaluronic acid
HAV	hepatitis A virus
HBV	hepatitis B virus
HBoV	human bocavirus
hCMV	human cytomegalovirus
HCV	hepatitis C virus
HE	hematoxylin-eosin
hFPT	human fetal progenitor tenocytes
HHV	human herpes virus
HIV	human immunodeficiency virus
HPV	human papilloma virus
HTLV	human T-cell lymphotropic virus
ISO	International Organization for Standardization
KIPyV	KI polyomavirus
MCB	master cell bank
MoA	mechanism of action
MRI	magnetic resonance imaging
MSC	mesenchymal stem cell
NO	nitric oxide
NSAID	non-steroidal anti-inflammatory drug
OECD	Organization for Economic Cooperation and Development
PBS	phosphate-buffered saline
PCB	parental cell bank
PDT	population doubling time
PDV	population doubling value
PEMF	pulsed electromagnetic field therapy
PRP	platelet-rich plasma
PTFE	polytetrafluoroethylene
RH	relative humidity
SAID	steroidal anti-inflammatory drug
SD	standard deviation
SR	Picrosirius red
SV40	simian virus 40
TEM	transmission electron microscopy
UK	United Kingdom
USA	United States of America
WCB	working cell bank
WUPyV	WU polyomavirus

## Appendix A

**Table A1:** Allocation table for rabbits included in the GLP preliminary safety study of hFPTs in a partial-thickness model of patellar tendon defect. GLP, good laboratory practice; hFPT, human fetal progenitor tenocytes.

Animal ID	Treatment Group	Operated Hindlimb
1	Test-item	Left
2	Test-item	Right
3	Test-item	Left
4	Reference item	Right
5	Reference item	Left
6	Reference item	Right

**Table A2:** Scoring table for macroscopic evaluation of the operated rabbit patellar tendons 6 weeks after intervention and treatment.

Parameter	Grading Scale with Corresponding Descriptions
Inflammation	0 (None); 1 (Mild); 2 (Severe)
Tissue adhesion	0 (Normal); 1 (Mild); 2 (Severe)
Fibrosis	0 (None); 1 (Mild); 2 (Severe)
Defect filling extent and quality	0 (Healthy tendon tissue with aligned fibers); 1 (Disorganized tendon tissue); 2 (Scar tissue, partial filling, or no filling)

**Table A3:** Scoring table for microscopic evaluation (i.e., 10 × enlargement) of sections from the operated tendons (i.e., two zones) 6 weeks after intervention and treatment.

Evaluation	Grading Scale with Corresponding Descriptions		
Hypercellularity ^1^ *[A – C]*	A (None); B (Mild); C (High)		
Vascularity ^2^ *[A – D]*	*Hypervascularity*	*Predominant Size*	*Transverse Vessels*
	A (No); B (Yes)	A (Small); B (Large)	A (None, Rare); B (Some, Many)
Inflammatory reaction *[A – D]*	A (None); B (Mild, some macrophages); C (Moderate, more macrophages, some foreign body cells); D (Severe, many foreign body cells, granulocytes)		
Foreign material *[A – C]*	A (None); B (Some, scattered); C (More, clustered)		
New matrix quality *[A – C]*	A (Tendon-like matrix); B (Pre-differentiated, immature matrix); C (Scar tissue, fibrosis)		
New matrix directionality *[A – C]*	A (Aligned fibers); B (Mildly disorganized fibers); C (No discernable directionality)		

^1^ Excluding inflammatory and vascular cells. ^2^ Grades are cumulative (i.e., 3 × A gives an overall grade of A, 2 × A and 1 × B gives an overall grade of B, 1 × A and 2 × B gives an overall grade of C, and 3 × B gives an overall grade of D).
