# Supplementary material for: Industrial Development of Standardized Fetal Progenitor Cell Therapy for Tendon Regenerative Medicine: Preliminary Safety in Xenogeneic Transplantation
Source: Biomedicines. 2021 Apr 3;9(4):380. doi: 10.3390/biomedicines9040380 (PMC8066015; doi:10.3390/biomedicines9040380)
Supplement: Supplementary file 1 [file biomedicines-09-00380-s001.pdf]

Supplementary figures

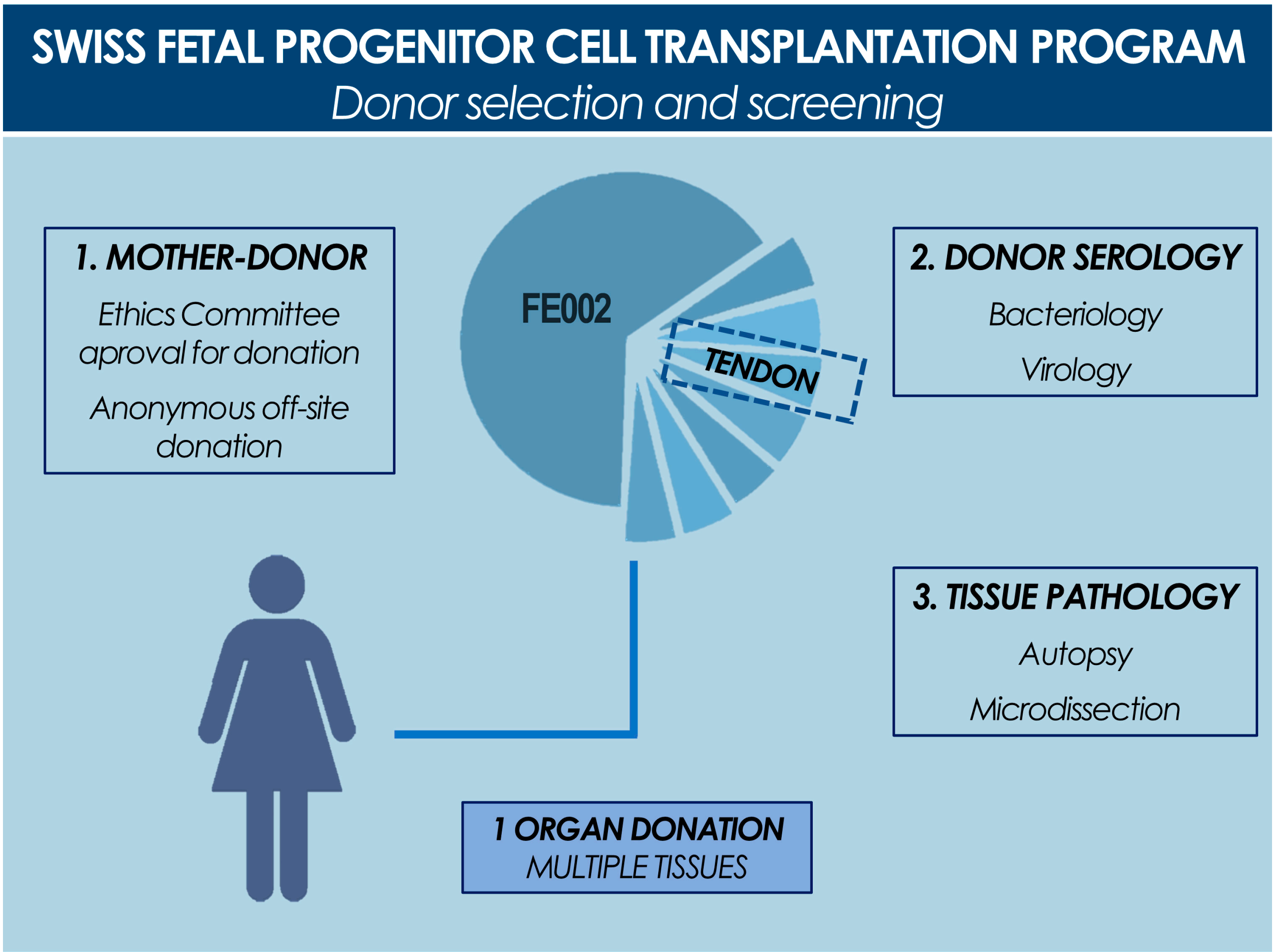

Figure S1. Schematic overview of the main steps in the Swiss FPC transplantation program. Specifically, the FE002 organ donation had been registered under the ad hoc Swiss federal FPC transplantation program, for inclusion of progeny cellular materials in a dedicated biobank, while assuring compliance with laws and regulations set forth by both framework programs. After appropriate and well-documented anonymous tissue procurement, FE002-Ten hFPTs and additional FPC types (e.g., isolated from bone, cartilage, intervertebral disc, lung, muscle, skin, etc.) were differentially isolated from the FE002 donation, following a validated protocol, which had been approved by the Vaud Cantonal Ethics Committee (CER, University Hospital of Lausanne, CHUV, Ethics Committee Protocol #62/07: "Development of fetal cell banks for tissue engineering", August 2007). FPC, fetal progenitor cells; hFPT, human fetal progenitor tenocytes.

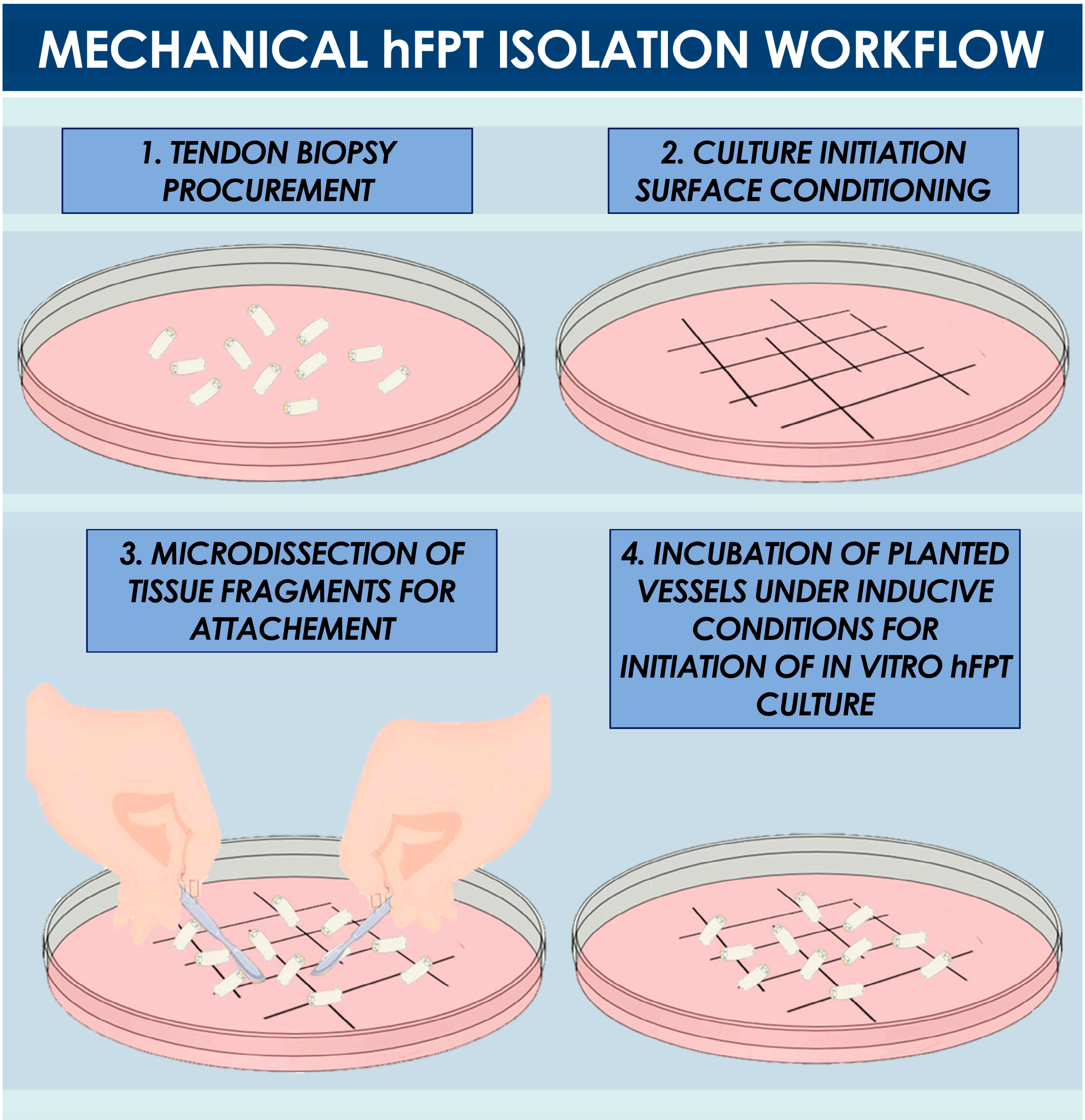

Figure S2. Schematic overview of fetal tendon biopsy processing for in vitro non-enzymatic isolation of hFPTs and primary culture initiation. Following primary FPC isolation from the FE002 donation under mechanical workflows, three specific cell types (i.e., dermal progenitor fibroblasts, hFPTs, and epiphyseal chondroprogenitors) were deposited in the European Collection of Authenticated Cell Cultures (ECACC, Porton Down, UK, N°12070301-FE002-SK2, 12070302-FE002-Ten, and 12070303-FE002-Cart, 2012) and at the Food Industry Research and Development Institute (FIRDI, Taiwan, N°BCRC 960460, N°BCRC 960461, and N°BCRC 960459, 2012). FPC, fetal progenitor cells; hFPT, human fetal progenitor tenocytes.

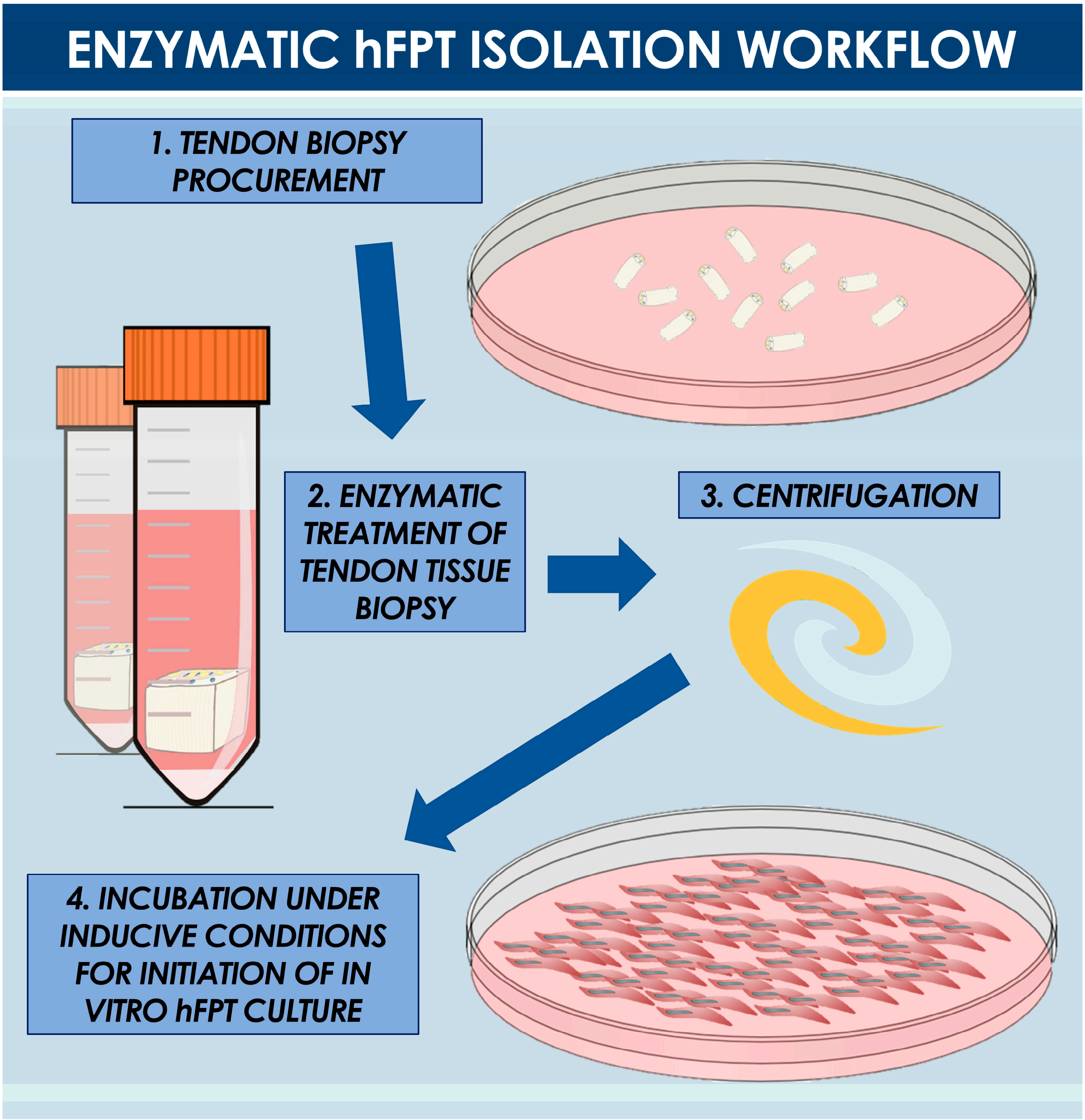

Figure S3. Schematic overview of fetal tendon biopsy processing for in vitro enzymatic isolation of hFPTs and primary culture initiation. After appropriate treatment with trypsin-EDTA (i.e., 0.25 % trypsin and 0.1 % ethylenediaminetetraacetic acid), dissociated hFPTs are centrifuged, collected, and plated in culture dishes for adherent cell proliferation initiation. EDTA, ethylenediaminetetraacetic acid; hFPT, human fetal progenitor tenocytes.

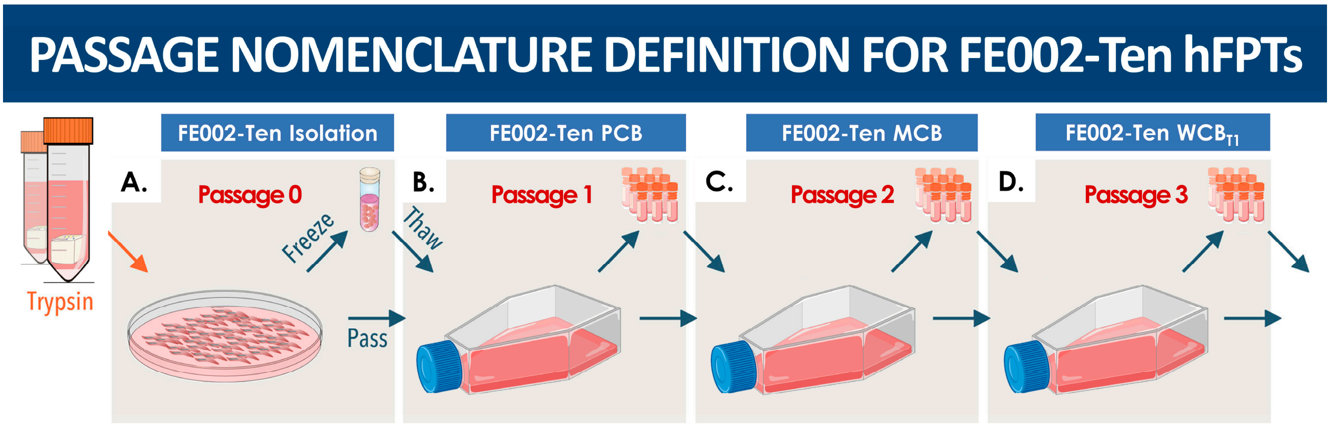

Figure S4. Schematic overview of enzymatic hFPT culture initiation and definition of in vitro passage numbers in relation with cell banking tiers. Therein, cells in PCB vials were defined as P1 in frozen state, becoming P2 upon thawing and subsequent culture expansion. (**A**) Initiation of FE002-Ten adherent culture following enzymatic cell isolation. (**B**) Expansion and harvest of Passage 1 cells. (**C**) Expansion and harvest of Passage 2 cells. (**D**) Expansion and harvest of Passage 3 cells. hFPT, human fetal progenitor tenocytes; MCB, master cell bank; PCB, parental cell bank; WCB, working cell bank.

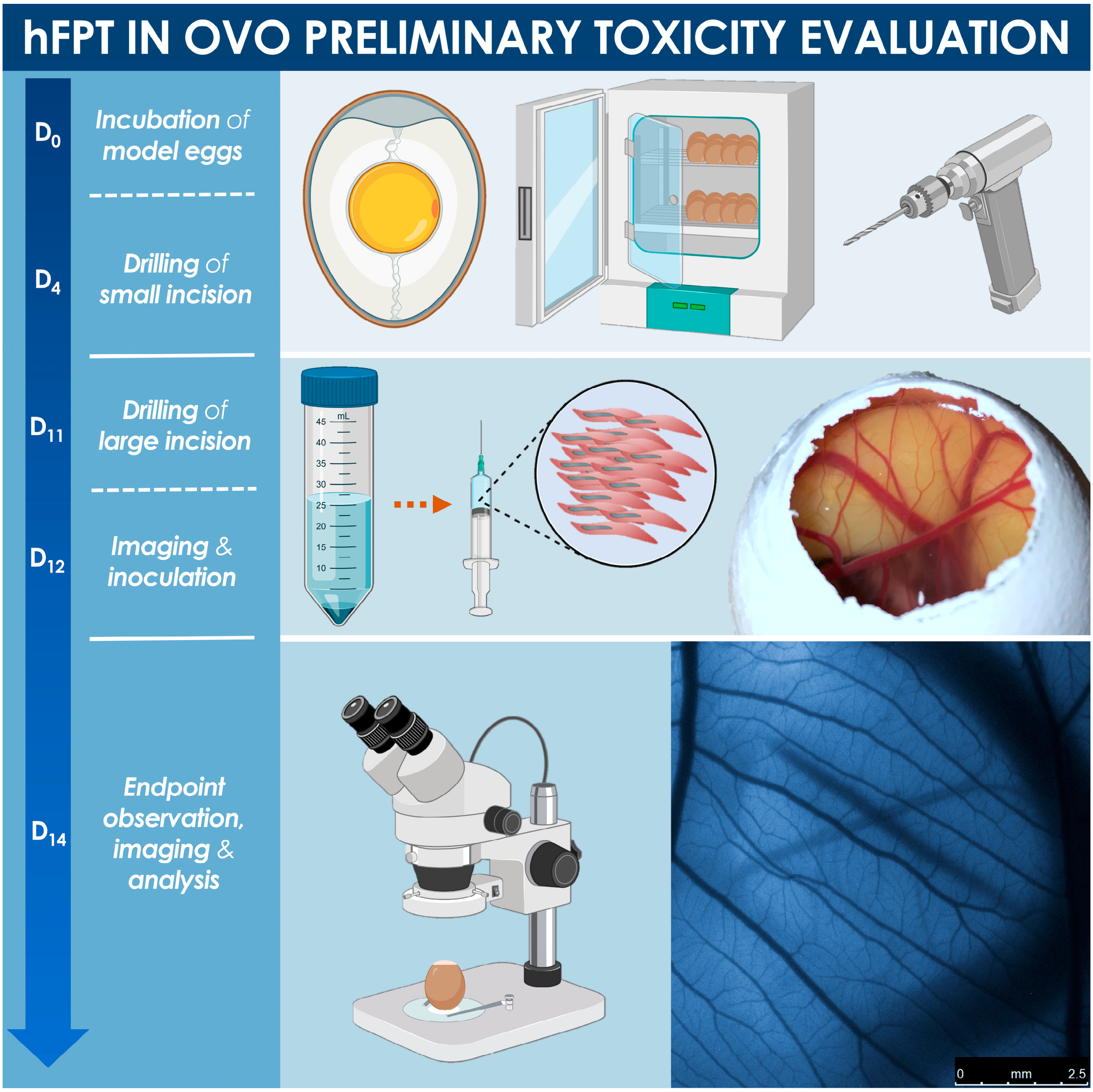

Figure S5. Overview of in ovo CAM assays for evaluation of hFPT toxicity on developing chicken embryos and effects on angiogenesis. Egg batches initially contained 60 eggs, providing at least ten eggs per condition at the time of endpoint analysis. Scale bar = 2.5 mm. CAM, chorioallantoic membrane; hFPT, human fetal progenitor tenocytes.

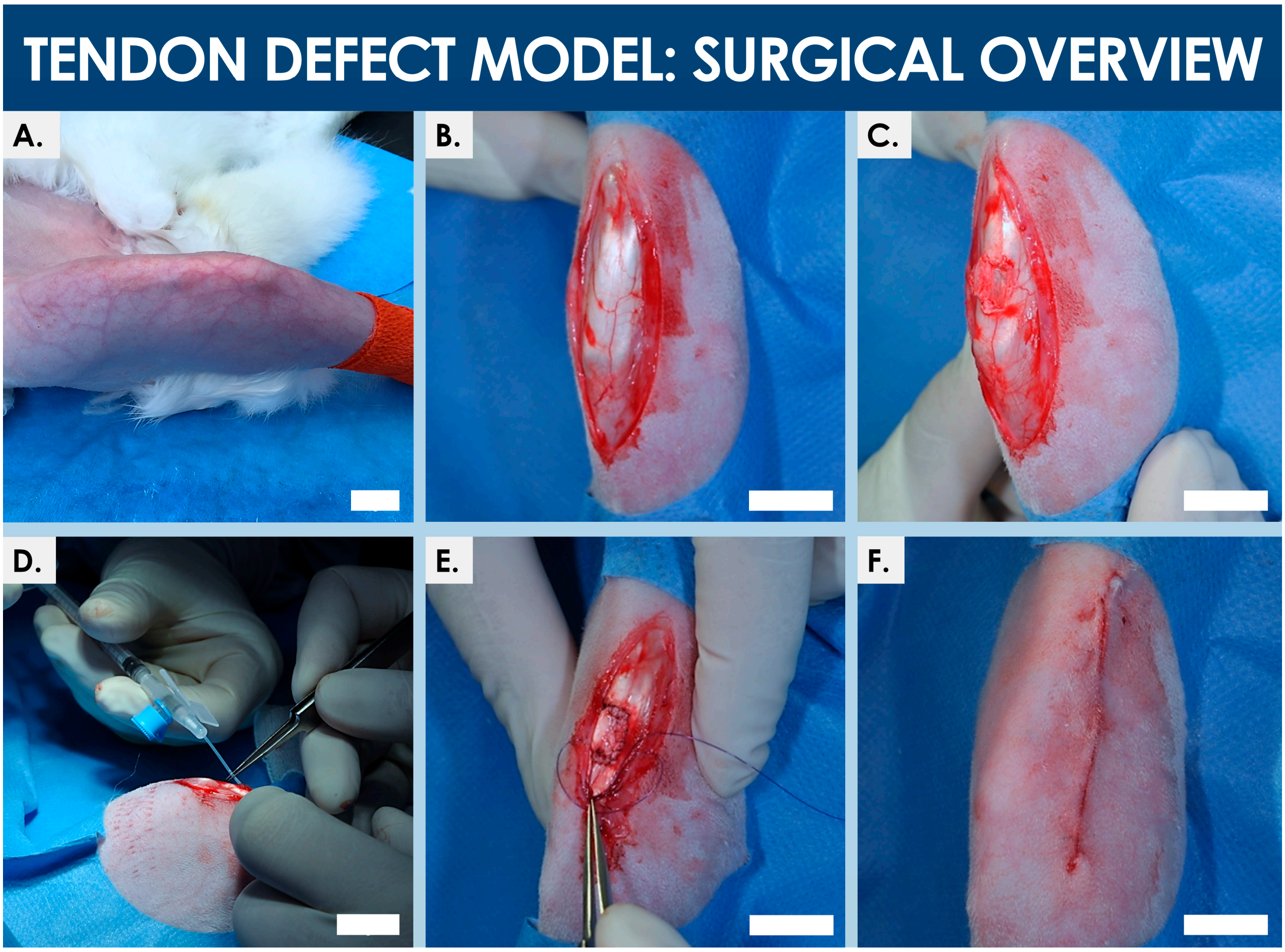

Figure S6. Overview of GLP surgery procedure for creation and treatment of a rabbit patellar tendon partial-thickness defect model. (**A**) One rabbit (e.g., rabbit N°2) hindlimb was shaved and the patellar tendon was localized. (**B**) An approximate three-centimetre-long incision was performed to access the patellar tendon. (**C**) A mid-thickness defect of approximately 6 mm long and 2 - 2.5 mm wide was created. (**D**) The flap was partially sutured, and the test-item or reference item were injected into the created defects (i.e., approximately 30 µL of product). (**E-F**) The tendon flap was completely closed and sutured in a continuous manner and the fascia and skin were closed with absorbable sutures. Scale bars = 10 mm. GLP, good laboratory practices.

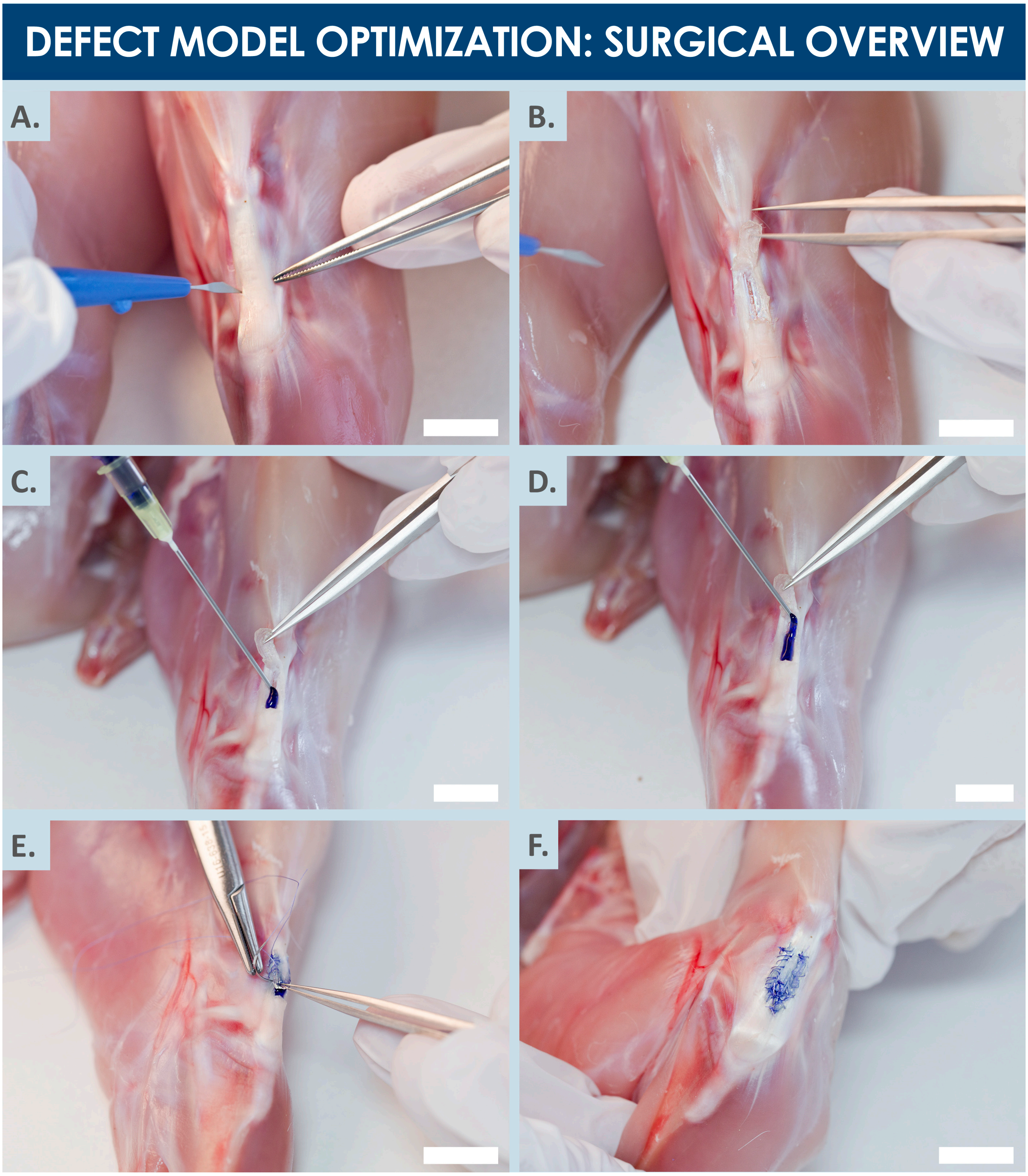

Figure S7. Photographic overview of the surgical optimization procedure performed on cadaver models for creating and treating the rabbit patellar tendon defect model. (**A**) Isolation of the tendon of interest. (**B**) Surgical creation of the tissue defect, with opening of a flap and scraping of the exposed tendon for cavity creation. (**C-D**) Administration of the placebo cell therapy product in the created cavity. (**E**) Closing and suturing of the flap. (**F**) Control flexion testing for verification of product leakage absence and suture resistance. Data were gathered during model adaptation phases performed on rabbit cadavers. Cadaver studies for surgical procedure adaptation enabled fine tuning of the protocol eventually used for the tendon defect model creation and investigation in the GLP preliminary safety study. To this end, the placebo cell therapy product was coloured with methylene blue, to follow the surgical procedure and evaluate the potential leaking of the implanted preparations. Scale bars = 10 mm. GLP, good laboratory practices.

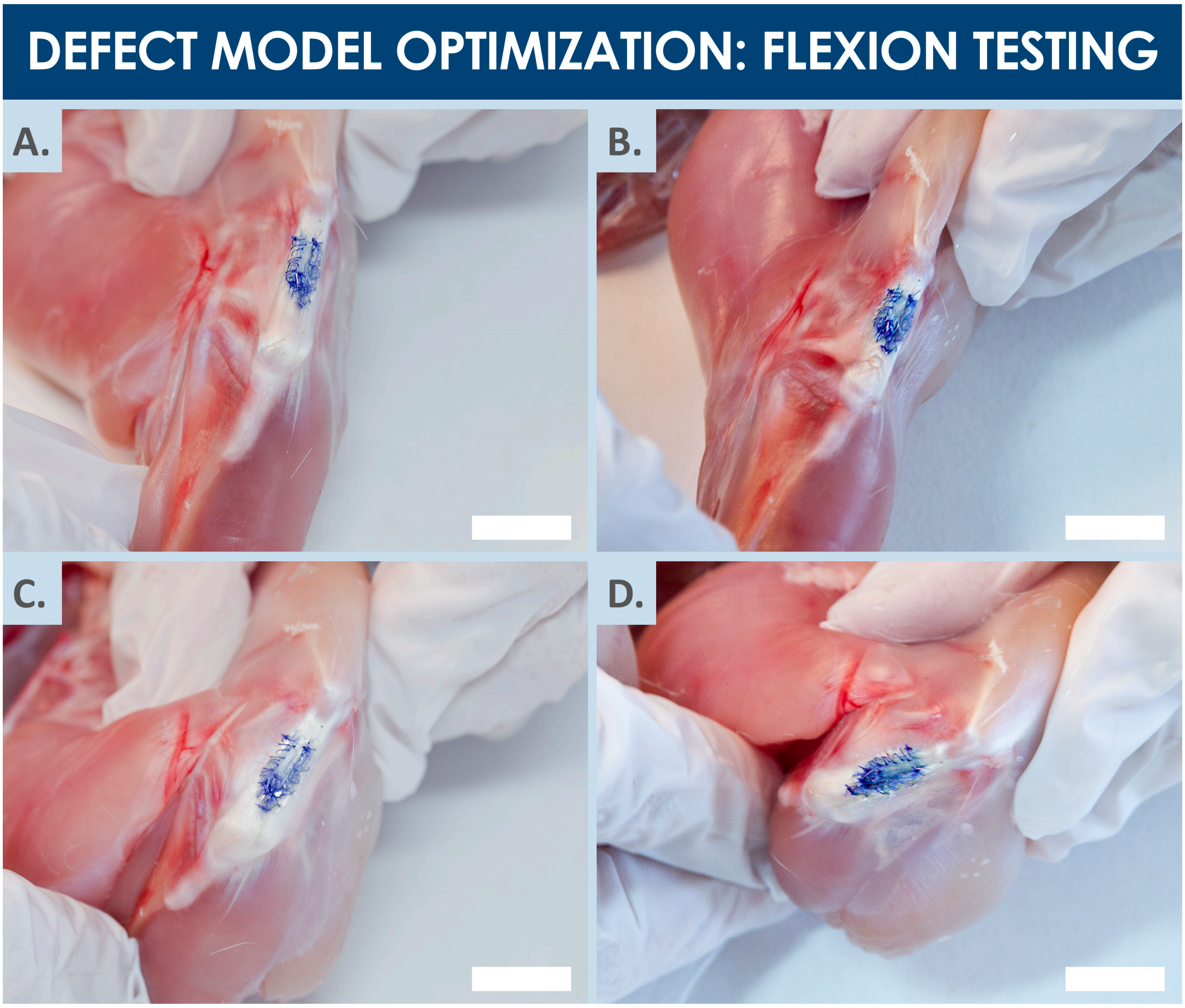

Figure S8. Photographic overview of the mechanical flexion testing of the operated and treated defect during surgical model optimization, for validation of tissue structural integrity and mobility maintenance after the intervention. (**A**) Flexion test with a 150-degree angle. (**B**) Flexion test with a 120-degree angle. (**C**) Flexion test with a 90-degree angle. (**D**) Flexion test with maximal flexion (i.e., around 35 degrees). Data were gathered during model adaptation phases performed on rabbit cadavers with coloured products in order to follow potential leaking of the treated artificial defects. Scale bars = 10 mm.

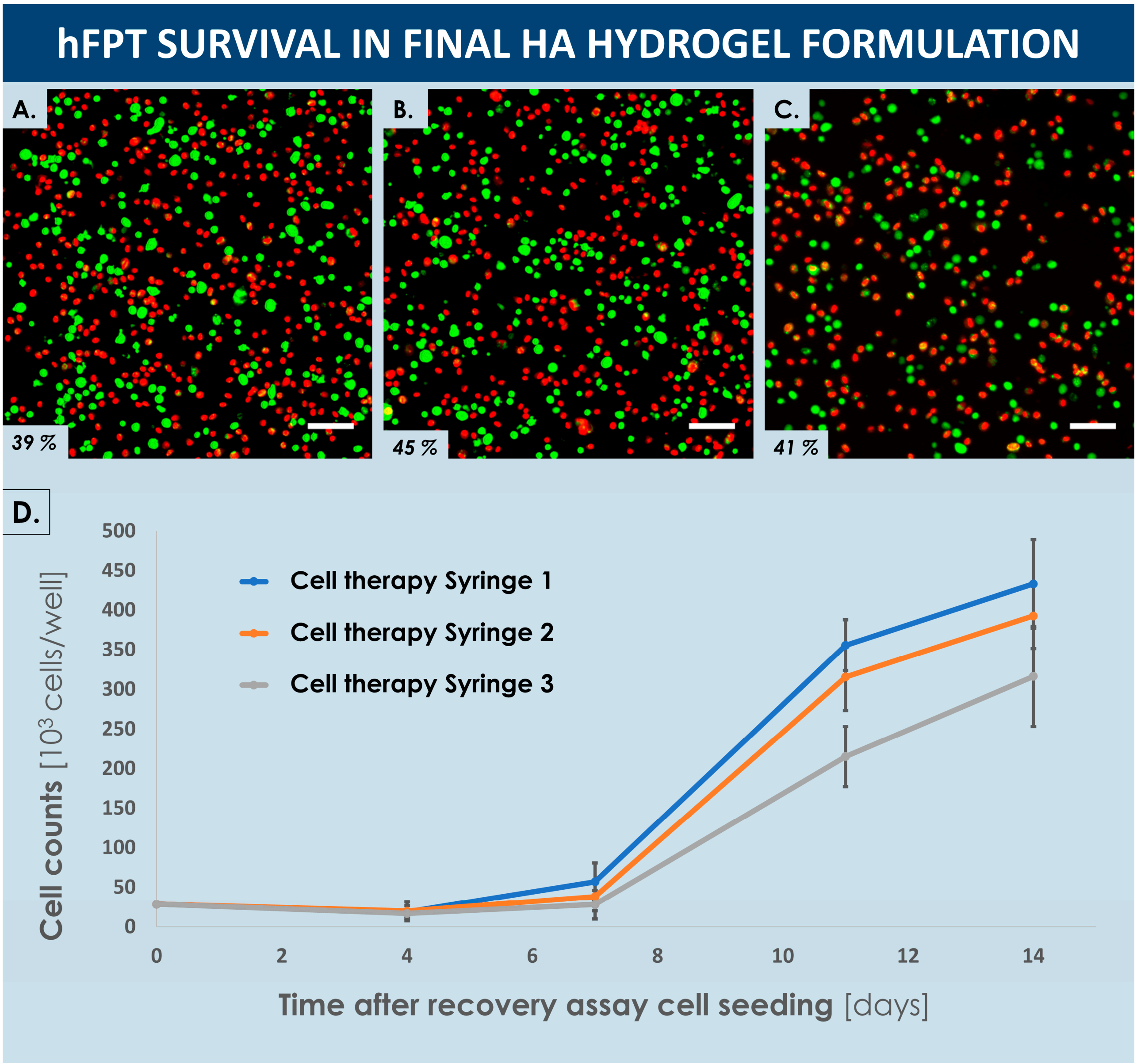

Figure S9. Survival and recovery of hFPTs from the spent test-items from the GLP pilot safety study. Preparation of hFPTs in HA hydrogel suspensions was rapid and efficient. The syringes were prepared prior to injection. All prepared products were observed to conserve their initial macroscopic properties between the end of manufacturing and clinical application. After injection, they were retained and stored at room temperature for up to eight hours, before being placed back at refrigerated temperature overnight. The LIVE/DEAD assay performed on the following day assured that a consistent proportion of viable cells had been injected (i.e., 39 %, 45 %, and 41 % viable cells, respectively). (**A-C**) Therapeutic cells remained viable in considerable and consistent proportions, as demonstrated by the presence of green dots in the LIVE/DEAD assay. Scale bars = 25 μm. (**D**) hFPTs suspended in HA and extruded from the syringes conserved the ability to attach and proliferate in standard culture conditions, as demonstrated in the recovery assays. The examined preparations did not display any signs of contamination during the recovery assays, performed without antibiotics in standard culture conditions. GLP, good laboratory practice; HA, hyaluronic acid; hFPT, human fetal progenitor tenocytes.

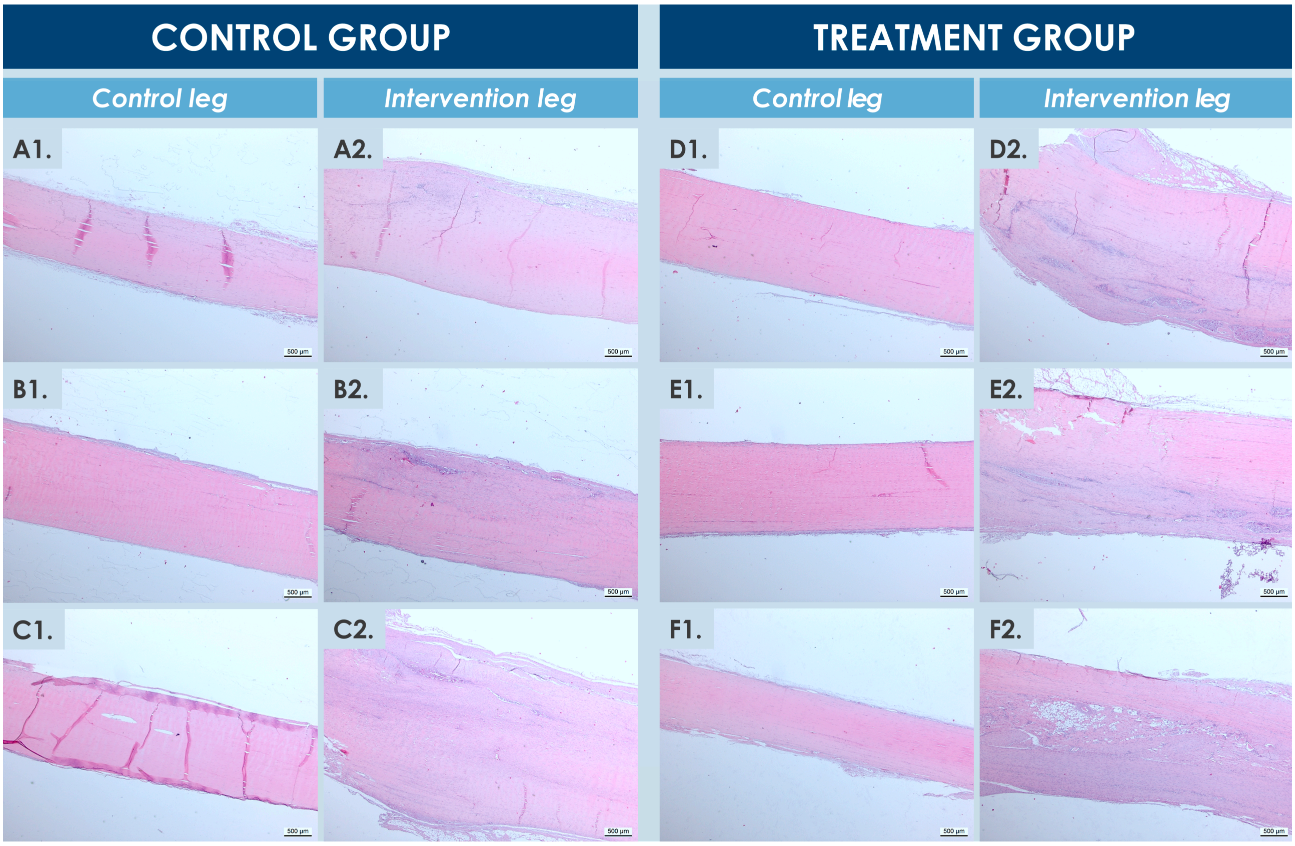

Figure S10. Photographic imaging of harvested rabbit patellar tendon sections from both groups (i.e., intervention legs and contralateral control legs, respectively) of the GLP pilot safety study, under 1.25 × optical magnification and after staining with hematoxylin-eosin. Data were analyzed and used for macroscopic assessment and scoring of the state of harvested tissues. Scale bars = 500 µm. GLP, good laboratory practices.

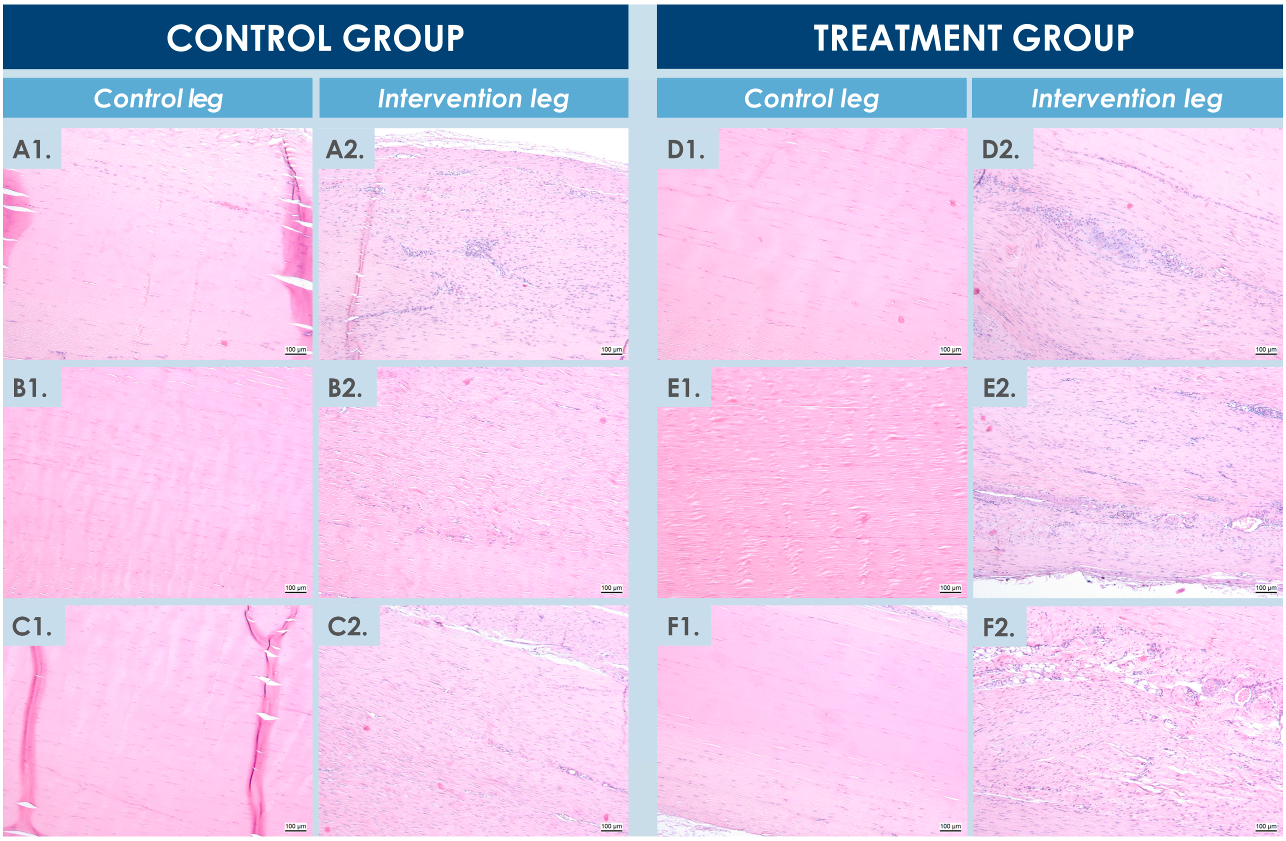

Figure S11. Photographic imaging of harvested rabbit patellar tendon sections from both groups (i.e., intervention legs and contralateral control legs, respectively) of the GLP pilot safety study, under 5 × optical magnification and after staining with hematoxylin-eosin. Data were analyzed and used for microscopic assessment and scoring of the state of harvested tissues. Scale bars = 100 µm. GLP, good laboratory practices.

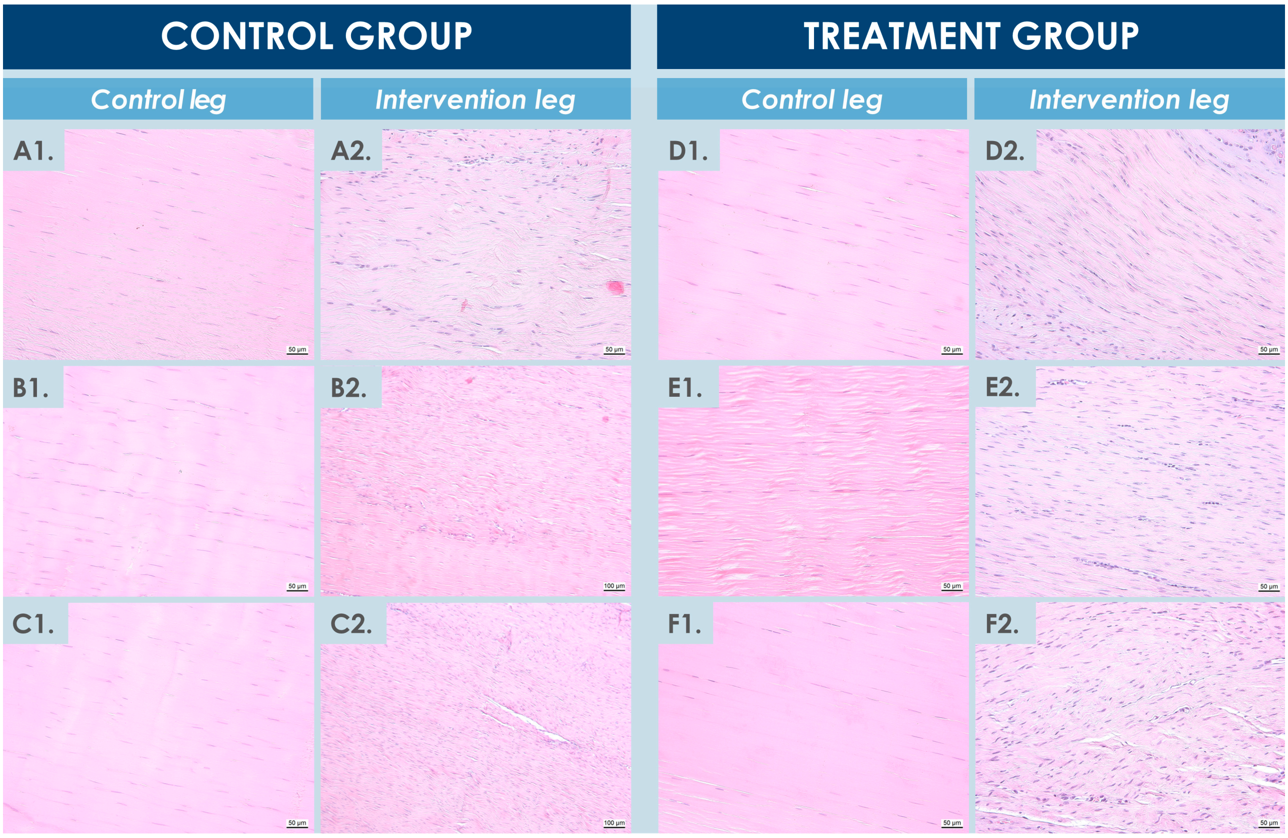

Figure S12. Photographic imaging of harvested rabbit patellar tendon sections at the defect area from both groups (i.e., intervention legs and contralateral control legs, respectively) of the GLP pilot safety study, under 10 × optical magnification and after staining with hematoxylin-eosin. Data were analyzed and used for microscopic assessment and scoring of the state of harvested tissues. Scale bars = 50 µm. GLP, good laboratory practices.

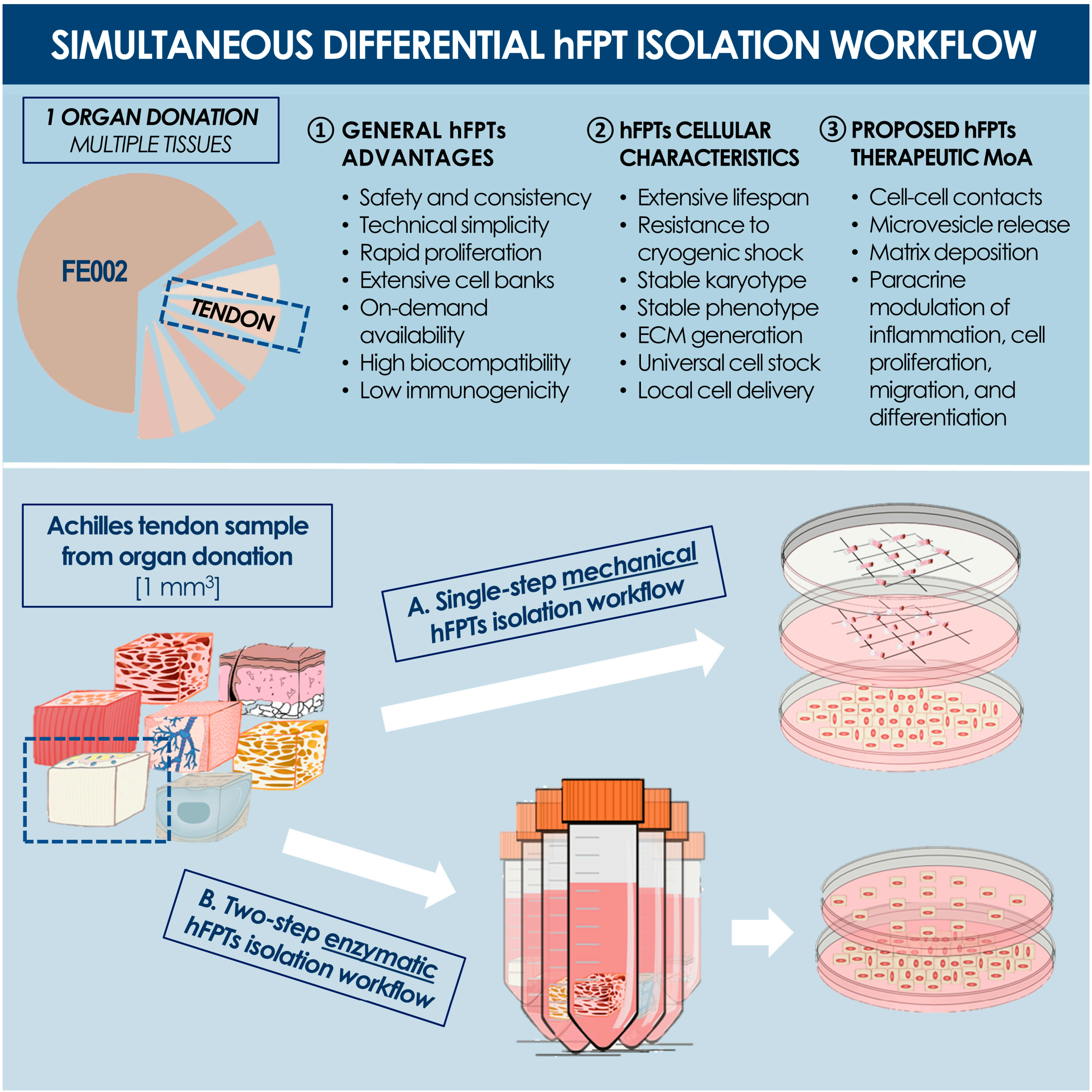

Figure S13. Overview of advantages related to the use of hFPTs as therapeutic agents and differential cell isolation workflows for initiation of adherent cell culture. From a single organ donation (i.e., FE002 donation, 2009) included in a specific transplantation program, various tissue biopsies (e.g., bone, cartilage, intervertebral disc, lung, muscle, skin, tendon, etc.) were simultaneously differentially treated for primary FPC isolation and in vitro culture initiation, parallelly using both enzymatic and non-enzymatic workflows. Optimized and standardized cell isolation procedures enable rapid and robust establishment of tissue-specific parental cell banks (PCB) to be valorized in diverse regenerative medicine applications. ECM, extra-cellular matrix; FPC, fetal progenitor cells; hFPT, human fetal progenitor tenocytes; MoA, mechanism of action.

Supplementary Tables

Table S1. Overview of characterization data gathered around the FE002-Ten hFPT cell type following initial establishment in 2009 [48,49]. CAM, chorioallantoic membrane; CD, cluster of differentiation; EOPCB, end of production cell bank; ECM, extracellular matrix; hFPT, human fetal progenitor tenocytes.

Cell type characteristics	Data, findings
Identity	"FE002-Ten" cell type, primary diploid hFPT cell type, isolated from Achilles tendon under an enzymatic or mechanical workflow<br>Fibroblast-like stable cellular morphology in adherent monolayer in vitro culture<br>Defined surface marker profile (i.e., D7-FIB$^+$, CD90$^+$, CD105$^+$, HLA-ABC$^+$, CD14$^-$, CD34$^-$, HLA-DP/DQ/DR$^-$, HLA-G$^-$)
Stability	Establishment and testing of EOPCBs at Passage 12<br>Normal 46X,Y karyotype, stable at least up to Passage 12<br>Lifespan > 12 passages with stable in vitro expansion kinetics<br>Resistance to adipogenic and osteogenic induction, cryogenic shock
Cytocompatibility	Cytocompatible with various hydrogel formulations (e.g., hyaluronan-based polymer gels)<br>Clinically relevant scaffold rheological behaviors of standardized transplant prototypes
Safety	Non-embryotoxic and non-angiotoxic in CAM model<br>Non-tumorigenic in soft agar transformation assays<br>Non-immunogenic, non-tumorigenic, and no slowing of tendon defect healing in rabbit model
Functionality	Potent and stable production of ECM<br>Stimulation potential toward adult tenocytes

Table S2. Clinically available options for tendon injury or degenerative disease treatment [56-59]. ESWT, extracorporeal shock wave therapy; HA, hyaluronic acid; NO, nitric oxide; NSAID, non-steroidal anti-inflammatory drug; PEMF, pulsed electromagnetic field therapy; PRP, platelet-rich plasma; SAID, steroidal anti-inflammatory drug.

Treatment Type	Description / Examples	Benefits	Limitations
Surgical interventions	Transplantation<br>Ablation<br>Sclerotherapy	Functional recovery<br>Scar tissue removal<br>Proven efficacy	Invasive procedures<br>Donor-site wounds<br>Technically demanding
Pharmacological treatment	Oral NSAIDs<br>Local NSAIDs<br>SAIDs injections<br>NO patches<br>HA injections<br>PRP injections<br>Growth factors<br>Cell therapies	Short-term pain relief<br>Relative functional gains<br>High innovation drive<br>Multiple products and drug classes<br>Ambulatory treatment	Long-term complications<br>Chronic analgesic use potential<br>Potential iatrogenesis<br>Potential safety issues<br>Palliative treatments
Physical therapy & device-mediated therapy	Specific exercises<br>ESWT<br>PEMF<br>Ultrasound<br>Iontophoresis<br>Low-level lasers<br>Phonophoresis<br>Hyperthermia	Short-term pain relief<br>Non-invasive<br>Individualized treatments<br>High innovation drive	Limited benefits<br>Lengthy recovery<br>Potentially expensive<br>High efficacy variability
Lifestyle intervention methods	Nutrition control<br>Weight loss<br>Physical re-education<br>Complementary medicine	Non-invasive methods<br>Relatively inexpensive	Lengthy recovery<br>Palliative treatment

Supplementary Methods

1. Primary hFPT Enzymatic Isolation and PCB Establishment

Anonymized tissue biopsies were processed by a fetal pathologist from the institutional Pathology Department (CHUV, Lausanne) after proper investigation, within the framework of the Swiss FPC transplantation program. Tissues were received in labelled containers, immersed in transport buffer (i.e., phosphate-buffered saline, PBS; NaCl 6.8 g/L, Na_2HPO_4 1.5 g/L, KH_2PO_4 0.4 g/L, N°100 0 324, Laboratorium Dr G. Bichsel AG, Switzerland). Tissues were rapidly processed for culture initiation or were stored at 4 °C until processing. Approximately 1 mm^3 of tendon tissue (i.e., from Achilles tendons) was isolated and rinsed thrice in conserved PBS (i.e., 1% penicillin-streptomycin, N°15140-122, Gibco™, UK). The available tissue was then dissected into < 0.03 mm^3 fragments and placed in sterile centrifuge tubes (50 mL, Falcon®, USA) before being covered with 5 mL of warmed (i.e., 37 °C) trypsin-EDTA (i.e., 0.25% trypsin and 0.1% ethylenediaminetetraacetic acid, Gibco™, USA) (Figure S3). After 15 min of incubation at 37 °C, enzymatic cell dissociation was ended by addition of liquid growth medium (i.e., Dulbecco's Modified Eagle Medium, DMEM, N°41966-029, Gibco®, USA; with 10% *v/v* clinical-grade fetal bovine serum, FBS, N°10101145, Invitrogen™, USA). Dissociation tubes were then centrifuged at 230 ± 10 × *g* at ambient temperature for 15 min, before resulting cellular materials were resuspended in warmed initial growth medium. Cell suspension titers (i.e., total and viable) were quantified on hemocytometers using Trypan Blue exclusion dye (Sigma-Aldrich®, USA). Cell suspensions (i.e., final medium volume of 10 mL) were then used to homogeneously seed six culture Petri dishes (N°353003, Falcon®, USA, Figure S3). Culture vessels were incubated in humidified incubators set at 37 °C ± 2 °C, 5% ± 0.5% CO_2, and 80% ± 10% relative humidity. Subsequently, growth medium was exchanged every 48 h. Culture vessels were microscopically examined on a phase contrast microscope at each medium exchange procedure, for confirmation of cell proliferation, adequate cellular morphology, and absence of microbial contamination. Once optimal initial banking confluency was attained (i.e., 50% for Passage 0 cells, Figure S3), cell cultures were rinsed twice with PBS before trypsinization (i.e., trypsin-EDTA, 0.05% trypsin-ethylenediaminetetraacetic acid, N°25300-054, Gibco™, USA). Following collection, cell suspension titers (i.e., total and viable) were quantified on hemocytometers using Trypan Blue exclusion dye. Suspensions were centrifuged at 230 ± 10 × *g* at ambient temperature for 15 min, before cells were resuspended in warmed complete growth medium (i.e., initial growth medium, with addition of L-glutamine, to reach a final concentration of 5.97 mM) and further distributed into one hundred T80 vented cell culture flasks (Nunc™, N°153732, USA) at a viable seeding density of (1.5 ± 0.1) × 10^3 cells/cm^2. Complete medium final volume was 15 mL/vessel, and cultures were incubated as described hereabove. Medium exchange procedures occurred every 48 h until 95% confluency was attained. After appropriate monitoring and controls, as described for the preliminary cultures, Passage 1 cells were harvested and resuspended in an appropriate cryopreservation medium (i.e., complete medium 50% *v/v*; FBS 40% *v/v*; dimethyl sulfoxide 10% *v/v*, DMSO N°D-2438, Sigma-Aldrich®, USA). Final viable cellular densities ranged from 2 × 10^6 to 10^7 viable cells/mL, and suspensions were homogeneously dispensed in labelled cryovials (1.8 mL cryovials, N°366656, Nunc™, USA). Initial controlled-rate freezing (i.e., -1 °C/minute rate) was performed using Nalgene® Mr. Frosty™ Cryo 1 °C Freezing Containers (Nalgene®, UK) and an ultra-low temperature (i.e., -80 °C) freezer. The next day, vial lots were transferred to the liquid nitrogen vapor phase of a quarantine Dewar storage tank. After proper validation and batch release, vial lots were further traceably stored in distinct secure storage tanks in several locations to mitigate risks of destruction. The considered cryopreserved material was defined as the enzymatically isolated FE002-Ten parental cell bank (PCB, Passage 1).

2. Primary hFPT Non-enzymatic Isolation and PCB Establishment

Anonymized tissue biopsies were processed by a fetal pathologist from the institutional Pathology Department (CHUV, Lausanne) after proper investigation, within the framework of the Swiss FPC transplantation program. Tissues were received in labelled containers, immersed in transport buffer (i.e., phosphate-buffered saline, PBS; NaCl 6.8 g/L, Na_2HPO_4 1.5 g/L, KH_2PO_4 0.4 g/L, N°100 0 324, Laboratorium Dr G. Bichsel AG, Switzerland). Tissues were rapidly processed for culture initiation or were stored at 4 °C until processing. Approximately 1 mm^3 of tendon tissue (i.e., from Achilles tendons) was isolated and rinsed thrice in conserved PBS (i.e., 1% penicillin-streptomycin, N°15140-122, Gibco™, UK), before transfer to culture Petri dishes (N°353003, Falcon®, USA), which had been scored in a checkerboard pattern (Figure S2). Biopsy fragments were further minced (i.e., < 0.03 mm^3 fragments) and attached along scored plastic regions (i.e., with 6-8 fragments/dish), favoring adherence and cell outgrowth in monolayers (Figure S2). Approximately 2 mL liquid growth medium (i.e., Dulbecco's Modified Eagle Medium, DMEM, N°41966-029, Gibco™, USA; with 10% *v/v* clinical-grade fetal bovine serum, FBS, N°10101145, Invitrogen™, USA) were

carefully dispensed around attached tissue fragments. Culture vessels were incubated overnight in humidified incubators set at 37 °C ± 2 °C, 5% ± 0.5% CO_2, and 80% ± 10% relative humidity. The following day, 10 mL of growth medium were carefully added to each dish, before cultures were incubated again. Subsequently, growth medium was exchanged every 48 h. Culture vessels were microscopically examined on a phase contrast microscope at each medium exchange procedure, for confirmation of cell emission and outward migration, adequate proliferative cellular morphology, and absence of microbial contamination. Once optimal initial banking confluency was attained (i.e., 50% for Passage 0 cells, Figure S2), cell cultures were rinsed twice with PBS before trypsinization (i.e., trypsin-EDTA, 0.05% trypsin-ethylenediaminetetraacetic acid, N°25300-054, Gibco™, USA). Following collection, cell suspension titers (i.e., total and viable) were quantified on hemocytometers using Trypan Blue exclusion dye (Sigma-Aldrich®, USA). Suspensions were centrifuged at 230 ± 10 × *g* at ambient temperature for 15 min, before cells were resuspended in warmed complete growth medium (i.e., initial growth medium, with addition of L-glutamine, to reach a final concentration of 5.97 mM) and further distributed into one hundred T80 vented cell culture flasks (Nunc™, N°153732, USA) at a viable seeding density of (1.5 ± 0.1) × 10^3 cells/cm^2. Complete medium final volume was 15 mL/vessel, and cultures were incubated as described hereabove. Medium exchange procedures occurred every 48 h until 95% confluency was attained. After appropriate monitoring and controls, as described for the preliminary cultures, Passage 1 cells were harvested and resuspended in an appropriate cryopreservation medium (i.e., complete medium 50% *v*/*v*; FBS 40% *v*/*v*; dimethyl sulfoxide 10% *v*/*v*, DMSO N°D-2438, Sigma-Aldrich®, USA). Final viable cellular densities ranged from 2 × 10^6 to 10^7 viable cells/mL, and suspensions were homogenously dispensed in labelled cryovials (1.8 mL cryovials, N°366656, Nunc™, USA). Initial controlled-rate freezing (i.e., -1 °C/minute rate) was performed using Nalgene® Mr. Frosty™ Cryo 1 °C Freezing Containers (Nalgene®, UK) and an ultra-low temperature (i.e., -80 °C) freezer. The next day, vial lots were transferred to the liquid nitrogen vapor phase of a quarantine Dewar storage tank. After proper validation and batch release, vial lots were further traceably stored in distinct secure storage tanks in several locations to mitigate risks of destruction. The considered cryopreserved material was defined as the mechanically isolated FE002-Ten parental cell bank (PCB, Passage 1).

3. Preclinical Toxicity Study of FE002-Ten hFPTs in a Standardized CAM Model

A standardized CAM model was devised to assess potential toxicity of FE002-Ten hFPTs and to study the in ovo effects thereof (Figure S5). Sixty fertilized chicken eggs (Animalco AG, Switzerland) were incubated at 37 °C in a humidified hatching incubator (FIEM srl, Italy) with apical extremities oriented downward and under cyclical rocking. Four days later, a one-millimeter diameter hole was drilled (Dentalwerk Buermoos drill, Germany) on the apical extremity of each egg, for detachment of chorioallantoic membranes from the outer shells. After parafilm-sealing, eggs were incubated again with apical extremities oriented upward. Six days later, two-centimeter openings were created (Dremel® engraver, Bosch Power Tools B.V., The Netherlands) on apical extremities, in order to remove the outer shell and expose the CAM membranes. Non-viable eggs were frozen at -20 °C overnight before being discarded. Thereafter, twelve-millimeter PTFE O-rings were placed on each CAM surface. Shell openings were then parafilm-sealed and eggs were incubated overnight. On the twelfth day after reception, CAMs were photographically recorded and test-items or reference items were dispensed in each O-ring. Test-items were constituted by 2.4 × 10^6 FE002-Ten hFPTs harvested at Passages 6 and 12, which were stored as dry pellets at -80 °C and which were extemporaneously reconstituted in 250 µL of normal saline. Cell pellets had been obtained from cultures, which had been rinsed with normal saline before centrifugation at 230 ± 10 ×*g* at ambient temperature for 15 min and discarding of supernatants. Reference items consisted in 250 µL of normal saline. After inoculation with test or reference items, the eggs were parafilm-sealed and incubated as described hereabove. Two days later (i.e., 14 days after reception), viability assessments were performed, before photographic imaging on a fluorescence stereomicroscope (Leica M205 FA, Leica Camera AG, Germany) was performed. Quality of angiogenesis was also comparatively assessed, along with observations on general egg and embryo appearance. Following assessment, eggs were frozen at -20 °C overnight before being discarded appropriately.

4. Preclinical Safety Study of FE002-Ten hFPTs in a Rabbit Model of Patellar Tendon Defect

4.1. Pilot GLP Safety Study Norms and Authorizations

A rabbit model was selected and adapted for the present study based on strong similarities between rabbit and human tendon tissues. The study was performed within the Vetsuisse Faculty in Zurich under the identification "MSRU0071 - Transplantation of human fetal progenitor tenocytes in a rabbit model for treatment of tendon injuries", with specified test-items "hFPT in hyaluronan-based gel". This safety study was initiated after proper internal ethical considerations had been taken into account and after validations had been obtained, in compliance with Principles of Good Laboratory Practice (GLP), as set forth by the Organization for Economic Cooperation and Development

(OECD), and adopted on November 26th, 1997. Investigation procedures had been adapted from specific norms and frameworks, namely the OECD Council Directive 93/42/EEC (June 1993) concerning medical devices, and ISO 10993 (i.e., Biological evaluation of medical devices) Parts 1, 2, 6, and 12. All experiments were conducted in compliance with Swiss federal laws on animal protection and welfare and had been duly authorized by the Zurich Cantonal Ethics Committee (License #ZH034/15).

4.2. Pilot GLP Safety Study Design

Six female New Zealand white rabbits were included in the in vivo GLP pilot safety study. At the time of surgery, animals presented an average age of 5.7 ± 0.7 months (i.e., values ranging from 5.2 to 6.6 months) and an average weight of 4.3 ± 0.7 kg (i.e., values ranging from 3.9 kg to 5.0 kg). The six included animals were randomized to form two treatment groups and were therefore separated in two groups of three animals. All animals were acclimatized appropriately for 14 days in test conditions. One group was operated and treated with the test-item (i.e., hFPTs suspended in HA) and the other group received the reference item (i.e., PBS diluted in HA). All rabbits were operated on one hindlimb (i.e., according to randomization, Table A1), the other hindlimb serving as an unoperated and untreated internal control (Figures 5 and S10 to S12). Following surgery and treatment, the rabbits were housed individually during the 24-72-h recovery period and in groups thereafter. Rabbits were sacrificed six weeks after surgery and the tendons of interest were harvested for evaluation and scoring, along with blood samples.

4.3. Cell-based Product Preparation and hFPT Viability Controls

Necessary materials and fresh cell cultures (i.e., FE002-Ten cells at Passage 6) were prepared under aseptic conditions in a class A laminar flow hood. Confluent hFPT cultures were rinsed with PBS, detached with TrypLE™ (Gibco™, The Netherlands) and transferred in centrifugation tubes, before addition of an equal volume of growth medium. Cells were enumerated on a haemocytometer and subsequently distributed into several tubes, with 28×10^6 cells/tube. Suspensions were then centrifuged at $230 \pm 10 \times g$ at ambient temperature for 15 min, before double rinsing with PBS for elimination of dissociation reagent and medium residues. For manufacture of test-items, 28×10^6 cells were resuspended in PBS to obtain a final volume of 175 μL. The resulting suspension was transferred to the body of a syringe (Omnifix-F 1 mL, B. Braun, Germany) with a micropipette. With the help of a female-female Luer adapter (Ark Plas, Flippin, AR, USA), 175 μL of a commercial HA hydrogel preparation (Ostenil Tendon®, TRB Chemedica AG, Germany) were added into the body of the syringe. A new Luer adapter was mounted on the syringe, with a new syringe on the other side, and the suspension was homogenized through serial passaging from one syringe to the other. Air bubbles were eliminated, the volume was corrected to 200 μL using HA, and a cap (Fresenius Kabi, Switzerland) was placed on the first syringe containing the product. The final cell concentration was therefore 2×10^6 cells/25 μL of product. Preparations were then individually conditioned in sterile packages. Product batches were stored at 4 °C for up to 72 h and were transferred to the operating room in a refrigerated box. At the time of clinical application, a sterile catheter (BD Venflon 22 G, 0.8 x 25 mm, BD, USA) was subsequently placed on the syringe for precise delivery into the tendon defects created in test subjects. For manufacture of reference items, the same procedure and materials were used, with the cell suspension being replaced by 175 μL of PBS.

Following clinical application, all syringes were conserved at room temperature for up to eight hours and subsequently placed back at refrigerated temperature. On the day following the clinical application, the remaining contents of syringes were used for cell survival analysis. Therefore, 50 μL were extruded from each syringe in a tube and were then diluted with 8 mL PBS. Volumes of 100 μL of cell suspension were then transferred to a 96-well plate and individually mixed with 100 μL LIVE/DEAD solution (Molecular Probes™, Life Technologies™, The Netherlands) prepared according to the manufacturer's instructions. After 30 min of incubation at room temperature and with protection from light, imaging was performed on an inverted microscope equipped for fluorescence (IX81, Olympus, Japan) and with a digital camera (iXon, Andor Technology, UK). Green and red channels were recorded separately, and the images were then analysed with Cell Profiler, to obtain the number of viable and dead cells. The identification of primary objects (i.e., live and dead cells) within both colour layers was set with a diameter of 10 to 120 pixels with automatic threshold strategy and distinction of clumped objects based on the shape, with dividing lines based on intensity. Each identification was validated manually to ensure accuracy of the method.

4.5. Surgical Technique for Patellar Tendon Defect Creation and Treatment

Three-centimetre-long incisions were created on the lateral face of the knee, extending to the medial face from one centimetre above to two centimetres below the knee joint space. A mid-tendon scalpel incision was made in the uppermost layer of the patellar tendon, creating a flap (i.e., approximately 6 mm long and 2 - 2.5 mm wide) (Figures S6C). The flap was lifted carefully, and a hollow space was created with a microscalpel blade within the underlying

tendon tissue, preserving the outer wall of the tendon. The flap was then partially suture-closed with absorbable Vicryl 6/0 (FS-3 needle and Polyglactin 910, Ethicon, USA) and the test or reference products were delivered into the defect cavity, filling it completely (i.e., approximately 30 µL of product, containing 2.4 × 10^6 cells in the treatment group). The tendon flap was then completely closed and sutured in a continuous manner with Vicryl 6/0, with products securely contained within the defect. The fascia was closed in layers with Biosyn 4-0 absorbable sutures (V-20 needle and monofilament Glycomer 631, Syneture, Medtronic, USA) in a simple continuous fashion. The skin was finally closed with Biosyn 4-0 and wounds were covered with standard compresses.

4.6. Post-operative Pharmacological Treatment and Prophylaxis

The post-operative recovery and observation period was set at two hours after the end of the surgical procedure. Post-operative analgesia consisted in buprenorphine (i.e., 0.01 mg/kg_{BW} s.c., Temgesic®, Indivior Schweiz AG, Switzerland) administered every six to eight hours after surgery, depending on visible signs of pain on the day of surgery and twice daily on the following days. Additionally, meloxicam (i.e., 1 mg/kg_{BW}, s.c., Metacam®, Boehringer Ingelheim, Germany) was administered once daily, when appropriate. Prophylactic post-operational antibiotic therapy consisted in enrofloxacin (i.e., 7.5 mg/kg_{BW}, s.c., Baytril®, Bayer AG, Switzerland) once daily on the day of surgery and for four days thereafter. Ranitidine was given as an antacid therapy twice perioperatively (i.e., before and after surgery, 1-2 mg/kg_{BW}, s.c. or i.v., Zantic®, GlaxoSmithKline SA, Switzerland). Operated hindlimbs were wrapped in appropriate bandages but were not immobilized.

4.7. In-life Animal Monitoring, Sacrifice, and Tissue Harvest

Animals were housed freely for six weeks with liberty of movement and access to water. Hay was provided ad libitum and rabbit pellets were provided twice daily. The rabbit status was controlled twice daily until sacrifice. Specific medical records were generated for each animal. Viability checks and clinical observations were performed two times on the day of surgery and at least twice daily thereafter. Body weight recording was performed once during acclimatization and once during the post-operative phase. At the defined six-week timepoint, the rabbits were sacrificed. Therein, animals were first sedated with alfaxalone (i.e., 3-5 mg/kg_{BW}, i.n.), matching the pre-operative dose. After sedation, an intravenous catheter was placed. A blood sample was collected at that moment. The animals were then euthanized with pentobarbital (i.e., 0.5 mL/kg_{BW}, i.v.). Sacrifice was confirmed by absence of heartbeat and pupillary reflex. Samples from tendon tissues of interest were harvested and prepared for further examination.

4.8. Blood Harvest and Analysis

Blood samples of approximately 0.5 mL in an EDTA K tube and 0.5 mL in a Li Heparin tube were collected from the central ear artery of each rabbit prior to operation and six weeks later, prior to sacrifice. The routine blood parameters were measured, with a special focus on leukocyte concentrations.

4.9. Macroscopic Assessment and Processing of Tendons

After sacrifice, the treated limbs were dissected, and a macroscopic evaluation was performed. The treated patellar tendons were cut at the insertion of the tuberositas tibiae and patella, inspected macroscopically, and documented using digital photography. Special focus was placed on key indicators such as local inflammation, tissue adhesion, fibrosis, and global tissue quality. A specific scoring method was devised, and scoring was performed to semi-quantitatively evaluate the tissues (Table A2). Harvested tendons were then transferred to 4% formalin, before being treated for histological analysis. The contralateral untreated patellar tendons were also harvested and placed in 4% formalin, to serve as untreated controls.

4.10. Histological and Immunohistochemistry Investigations of Harvested Tendons

Both operated and contralateral patellar tendons had been fixed in 4% formalin for up to two days, then placed in 75% ethanol for up to three days before embedding in paraffin. The operated tendons and the contralateral patellar tendons were embedded within the same block for each animal, along with a reference lung tissue sample in order to recognize the operated tendon within the block, as well as the proximal-distal orientation of the tendons. Sections of five µm were cut in the sagital plane following the direction of tendon fibers and were stained with hematoxylin-eosin, Alcian Blue, Picrosirius Red, and von Kossa stains. Tendon healing was assessed by studying tendon morphology at the original defect site with a phase contrast microscope (Leica DMR, Leica Microsystems, Germany). A gross evaluation was performed with a 5 × objective to evaluate the overall quality of the repair site (Figure S11). The defect location, size, and edges were observed, as well as the reactivity of the surface (i.e., graded 0 for no reaction, 1 for mild reaction, and 2 for severe reaction, Table A2). The presence of zones with structural abnormalities (i.e., disorganization,

fat tissue presence, foreign material inclusion, hypervascularity, hypercellularity, calcifications) was also recorded. Two zones were then selected within the defect areas for a microscopic evaluation. When remnants of biomaterial were suspected, this zone was chosen around the biomaterial, and the other zone was chosen in a region distal from it. The regions comprising suture materials were not selected as zones of evaluation. Tendon tissues were then evaluated with a 10 × objective and scoring was performed based on Table A3 (Figure S12). In situ hybridization was finally performed on tissue sections for the detection of human DNA ALU repeats. A camera (Leica DFC320, Leica Microsystems) was used for acquisition of photographic images of interest.
